# Recent Advances in the Detection of Food Toxins Using Mass Spectrometry

**DOI:** 10.1021/acs.chemrestox.3c00241

**Published:** 2023-12-07

**Authors:** Vishal Ahuja, Amanpreet Singh, Debarati Paul, Diptarka Dasgupta, Petra Urajová, Sounak Ghosh, Roshani Singh, Gobardhan Sahoo, Daniela Ewe, Kumar Saurav

**Affiliations:** † University Institute of Biotechnology, Chandigarh University, Mohali, Punjab 140413, India; ‡ University Centre for Research & Development, Chandigarh University, Mohali, Punjab 140413, India; § Department of Chemistry, University Institute of Science, Chandigarh University, Mohali, Punjab 140413, India; ⊥ Amity Institute of Biotechnology, AUUP, Noida, Uttar Pradesh 201313, India; ¶ Material Resource Efficiency Division, CSIR-Indian Institute of Petroleum, Dehradun 248005, India; # Laboratory of Algal Biotechnology-Centre Algatech, Institute of Microbiology of the Czech Academy of Sciences, Třeboň 379 01, Czech Republic

## Abstract

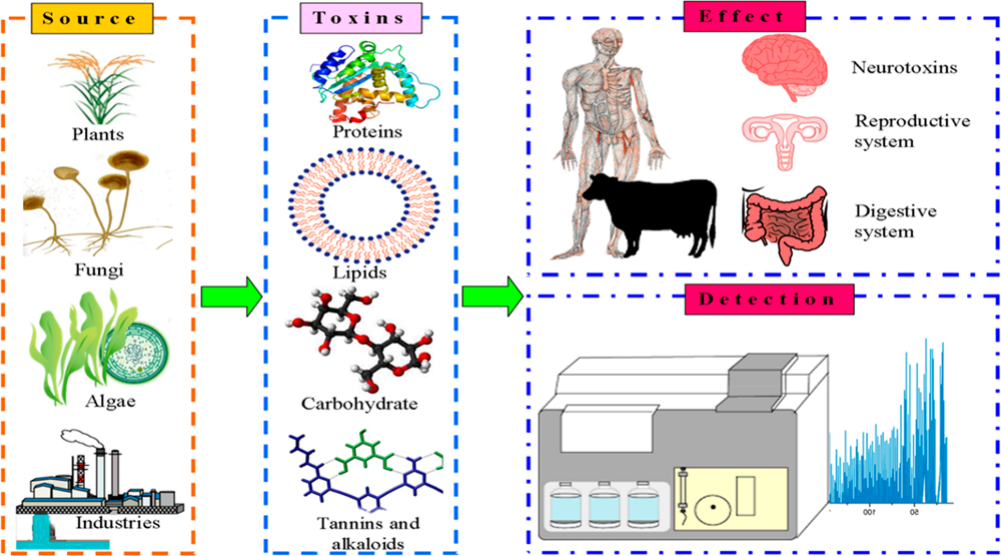

Edibles are the only source of nutrients and energy for humans. However, ingredients of edibles have undergone many physicochemical changes during preparation and storage. Aging, hydrolysis, oxidation, and rancidity are some of the major changes that not only change the native flavor, texture, and taste of food but also destroy the nutritive value and jeopardize public health. The major reasons for the production of harmful metabolites, chemicals, and toxins are poor processing, inappropriate storage, and microbial spoilage, which are lethal to consumers. In addition, the emergence of new pollutants has intensified the need for advanced and rapid food analysis techniques to detect such toxins. The issue with the detection of toxins in food samples is the nonvolatile nature and absence of detectable chromophores; hence, normal conventional techniques need additional derivatization. Mass spectrometry (MS) offers high sensitivity, selectivity, and capability to handle complex mixtures, making it an ideal analytical technique for the identification and quantification of food toxins. Recent technological advancements, such as high-resolution MS and tandem mass spectrometry (MS/MS), have significantly improved sensitivity, enabling the detection of food toxins at ultralow levels. Moreover, the emergence of ambient ionization techniques has facilitated rapid in situ analysis of samples with lower time and resources. Despite numerous advantages, the widespread adoption of MS in routine food safety monitoring faces certain challenges such as instrument cost, complexity, data analysis, and standardization of methods. Nevertheless, the continuous advancements in MS-technology and its integration with complementary techniques hold promising prospects for revolutionizing food safety monitoring. This review discusses the application of MS in detecting various food toxins including mycotoxins, marine biotoxins, and plant-derived toxins. It also explores the implementation of untargeted approaches, such as metabolomics and proteomics, for the discovery of novel and emerging food toxins, enhancing our understanding of potential hazards in the food supply chain.

## 1 Introduction

Serious health hazards and outbreaks resulting from food spoilage are major concerns of food safety worldwide.^1^ Due to significant biological activity and poor detection by conventional testing techniques, food toxins among other contaminants constitute a serious risk to the public’s health.^2,3^ Food toxins are compounds that can contaminate different food products during manufacturing, processing, transit, or storage.^4^ According to Fletcher and Netzel, these poisons can come from a variety of sources including fungi, bacteria, algae, plants, and animals.^5^ Mycotoxins produced by molds, marine biotoxins from toxic algal blooms, and plant-derived toxins like alkaloids and glycoalkaloids are all well-known examples of food toxins.^6,7^ Mycotoxins are mainly produced by toxigenic fungal species belonging to the genera of *Fusarium*, *Aspergillus*, and *Penicillium*.^8^ These mycotoxins pose a challenge to food safety because they can contaminate food products even when good storage and processing protocols are employed for food safety. Mycotoxin contamination accounts for the major cause of food borne diseases as reported by the World Health Organization (WHO). Recent studies performed by the European Commission revealed that 80% of the samples were contaminated with at least one mycotoxin.^9^ Perusal of literature revealed that most of the mycotoxins are chemically and thermally stable; therefore, they can survive under storage, processing, and even cooking.^10^ It has been observed that crops that are stored for more than a few days become probable targets for the growth of fungi and mycotoxin formation. These mycotoxins can affect a variety of food commodities such as dried fruits, coffee, spices, nuts, cereals, oil seeds, fruits, spices, cocoa, beans, etc.^11^ Apart from mycotoxins, other microorganisms like bacteria, algae, and even plants also produce such metabolites that are equally toxic and lethal. Bacteria contamination is mainly attributed to poor hygiene and cleanliness and common uncleaned and poorly cooked meat and vegetable-based foods, especially fermented foods. Nem Chua fermented food prepared from pork sausage in Vietnam has the possibility for *Staphylococcus aureus* contamination, and New Zealand mussel (*Perna canaliculus*) traditional fermented food of New Zealand is usually contaminated with *Clostridium botulinum*.^12^ Likewise, aquatic and marine food like Shellfish, fish, and even water are contaminated with algal biotoxins.^13^ In the case of plants, some normal metabolites produced for various purposes like natural defense and stress tolerance act as toxins for other organisms. Cyanogenic glycosides in almonds, and summer fruits, furocoumarins in citrus fruits, and lectins in beans are some of the common examples of phytotoxins.^14^ Besides, some newly emerging chemicals include perchlorate, flame retardants, halo compounds, packaging materials, petrochemicals residues, healthcare products traces, and microplastics.^15^ The use of contaminated cereals, grapes, and barley used to produce wine and beer products and their consumption are the main causes of toxicological effects in human beings (Table 1).

**Table 1:** Various Food Toxins from Various Sources

Class	Toxin’s name	Source	Effect	Ref
Mycotoxins	Aflatoxin	*Aspergillus flavus* and *A. parasiticus*	Liver failure, cirrhosis	(16)
	Lysergic acid (ergot alkaloids)	*Claviceps purpurea*	Ergotism, vasoconstriction, uterine contraction	(17)
	Fumonisins B1 and B2	*Fusarium verticillioides* and *Fusarium proliferatum*	disruption of sphingolipid metabolism, leuko-encephalomalacia	(18)
	Ochratoxin A	Aspergillus and Penicillium	Carcinogenic, immunotoxic mutagenic, nephrotoxic, and teratogenic	(19)
	Patulin	*Aspergillus*, Byssochlamysand *Penicillium*	Teratogenic, carcinogenic and mutagenic	(20)
	Zearalenone	*Fusarium graminearum*, *F. culmorum*, *F. crookwellense*, *F. poae*, *F. semitectum*, and *F. equiseti*	Hepatotoxicity, immunotoxicity, reproductive toxicity	(21)
	Tentoxin	*Alternaria*	Genotoxic, mutagenic, and carcinogenic	(22)
Bacterial toxins	Cholera toxins	*Vibrio cholerae*	diarrhea	(23)
	Enterotoxins	*Staphylococcus epidermidis*	Toxic shock syndrome	(24)
	Shiga toxins	*Escherichia coli*	Gastrointestinal complications	(25)
	Botulinum toxins	*Clostridium botulinum*	Neurotoxic	(26)
	Cereulide	*Bacillus cereus*	Dysfunction of liver, pancreatic islet, intestines, brain,	(27)
Marine biotoxins	Saxitoxin	Cyanobacteria and dinoflagellates	Neurotoxin, paralysis	(28)
	domoic acid	Diatoms	Neurotoxin	(29)
	Azaspiracid	*Azadiniumpoporum*	Diarrheic shellfish poisoning	(30)
	Brevetoxin	*Karenia brevis*	Immunotoxicity	(31)
	okadaic acid	*Halichondriamelanodocia* and *Halichondriaokadai*	Diarrhea, nausea	(32)
Plant-based toxins	Cyanogenic glycosides	Almonds, cassava, pome fruit, stone fruit	Tissue damage	(33)
	Furocoumarins	Citrus fruits	Skin cancer	(34)
	Ptaquiloside	Bracken ferns	Carciogenic	(35)
	Dehydropyrrolizidine	*Cyanoglossum*, *Senecio*, *Echium*, *Crotalaria*, *Heliotropium*, *Symphytum*, *Trichodesma*	Carcinogenic	(36)

The use of contaminated cereals, grapes, and barley for the production of wine and beer products is the cause of toxicological effects in human beings. Consumption of contaminated meat and milk-based products is another route for these toxins to enter the human food chain. Further, the abusive use of drugs in livestock, animal waste pollution, and the use of industrial wastewater for irrigation are also responsible factors for the entry of these contaminants into the food chain.^37^ Conventional methods for the detection of food toxin include immunoassays and chromatographic techniques, which, while effective, have certain limitations.^38^ Immunoassays, for instance, can be sensitive but may give false results if structurally related compounds are present in the testing matrix.^10^ Chromatographic techniques, on the other hand, require complex sample preparation and longer analysis times.^39^ In contrast, significant advancements in analytical techniques have revolutionized the field of food safety, and one such breakthrough is the application of mass spectrometry (MS) for the rapid and sensitive detection of food toxins. It is highly sensitive and provides selectivity and capability to handle complex mixtures, making it an ideal tool for the detection and characterization of food toxins.^40,41^ This led to the selection of the MS-based approach as the first choice tool among researchers, regulatory agencies, and food industries to ensure the safety and quality of the food supply chain.^41−43^

Recent advances and innovations in instrumentation, such as the development of high-resolution mass spectrometry (HRMS) and tandem mass spectrometry (MS/MS), have significantly improved sensitivity and selectivity, allowing for the detection of food toxins at ultralow levels.^44^ Additionally, several ionization methods allow rapid in situ analysis, reducing the time and resources required for analysis.^45,46^ Further, advancement in the field of untargeted metabolomics and several web-based repositories of metabolites allows for the detection of not only the known toxin but also the unknown variants of toxins, broadening our understanding of potential hazards in the food supply chain.^47,48^ The current review summarizes the overview of available detection techniques of food toxins and then further elaborates on the MS-based approaches, their benefits and drawbacks, and how they are used in various food matrices. We will also go over the difficulties in applying MS-based techniques to routine food safety monitoring as well as the potential of this technology to protect public health and global food security.

## 2 Conventional Methods for the Detection of Food Toxins

**Figure 1:**
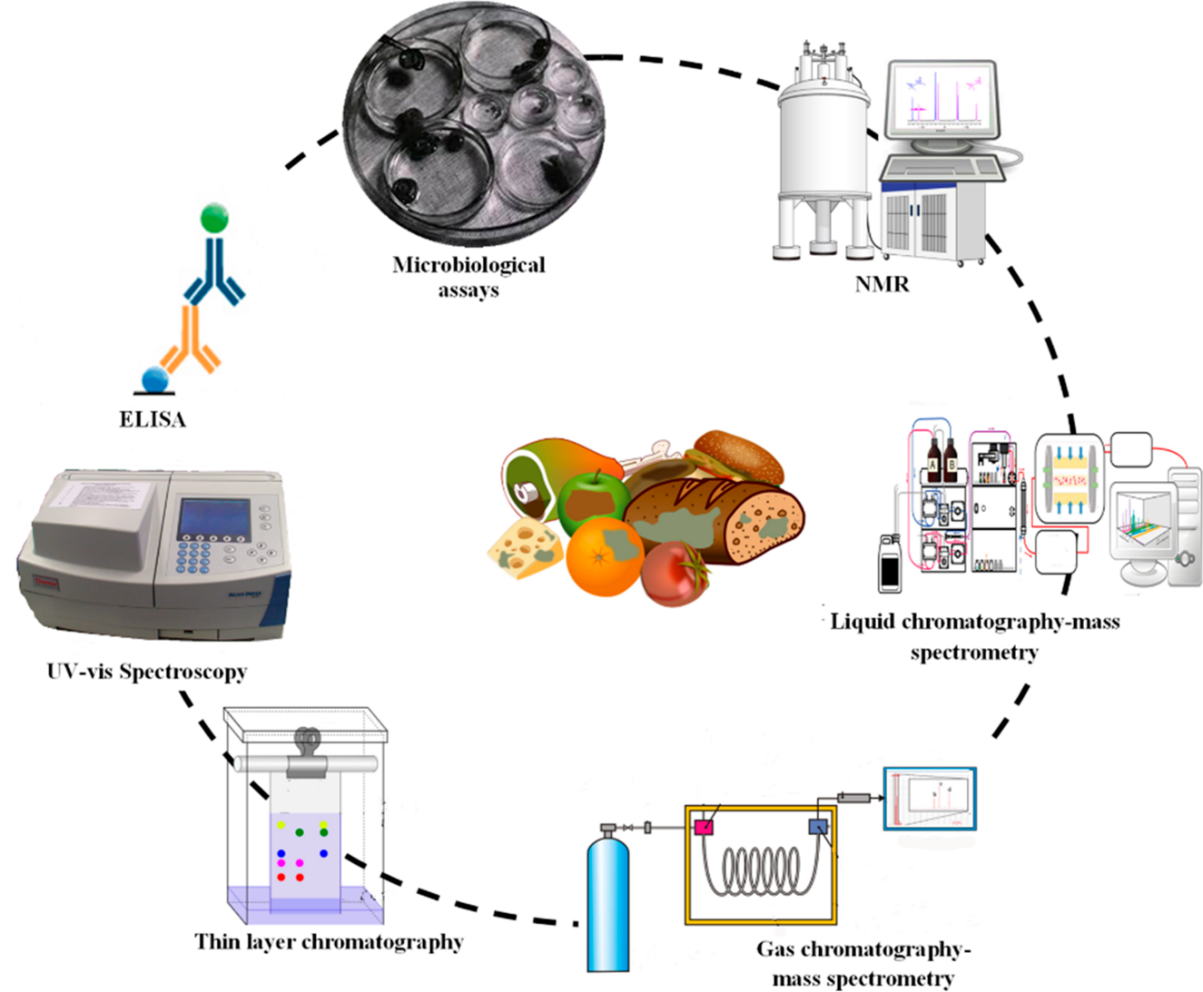
Schematic representation of various methods used for the detection of food toxins.

There are several specific and well-established conventional methods used on a regular basis for food safety assessment and regulation. These methods are often specific, but they may have limitations in terms of sensitivity, speed, and ability to detect a wide range of toxins (Figure 1). Some of the most common conventional methods for the detection of food toxins are mentioned below.

### 2.1 Immunoassays

The most commonly used immunoassay methods in the field of food toxin detection are enzyme-linked immunosorbent assays (ELISA) and lateral flow immunoassays. These methods are very rapid and help with easy detection. Samples are allowed to interact with either labeled enzymes or antibodies, thus helping in the detection. However, compounds with similar core structures or nontoxic analogs can provide false positive results. Phycotoxins like Okadaic acid, Yessotoxin, Pectenotoxin, Azaspiracid, Cyclic imines, Palytoxin, Domoic acid, Saxitoxin, Microcystin, and Cylindrospermospsin; mycotoxins like Aflatoxin B1, Deoxynivalenol, Fumonisin B1 Zearalenone, and T-2; and bacterial toxins like *Clostridium perfringens* α, β, and ε toxin, *Staphylococcal* enterotoxins A, B, C, and E, botulinum toxins, and *Escherichia coli* enterotoxins^49−51^ are detected using immunoassay methods.

### 2.2 Chromatographic Techniques

Thin-layer chromatography (TLC), high-performance liquid chromatography (HPLC), and gas chromatography (GC) are commonly used to separate and quantify food toxins. Among various seafood-originated toxins, Domoic acid, paralytic shellfish toxins, and Aflatoxin B1 can be easily detected by HPLC and TLC.^52−55^ Among all, TLC is a simple technique in which food toxins are chromatographed on a plate layered with a thin layer of stationary phase using different mobile phase solvents, aiding the separation and visualization of the toxins. On the other hand, HPLC and GC are more advanced ways to separate toxins depending on various principles, enabling us to separate and quantify different food toxins. HPLC and GC analysis needs often include a complex protocol for sample preparation, which is one of the major limitations in the application of these techniques.

### 2.3 Spectroscopic-Based Techniques: UV–visible and Fluorescence Spectroscopy

Each toxin possesses a different core structure with different motifs and thus absorbs light at a particular wavelength, enabling its detection if monitored at their respective wavelength. This principle is harnessed for the detection of food toxins such as aflatoxins and can be measured with UV-fluorescence spectroscopy.^56^ It was suggested that if a sample is showing response at 400 and 550 nm with respect to 365 and 730 nm excitation wavelengths, it is supposed to be contaminated with aflatoxins. Singh et al.^57^ also reported that aflatoxin B1 and ochratoxin A have maximum absorption (λ_max_) at 365 and 380 nm. Both UV–visible and fluorescence spectroscopic techniques are easy to handle and cost-effective. Moreover, fluorescence spectroscopy has a high sensitivity for the detection of food toxins. Despite these advantages, there are certain toxins whose absorption and emission wavelengths may not be very selective and specific, making one of the major limitations in their detection by this technique. Other spectroscopic methods like nuclear magnetic resonance (NMR) spectroscopy are also used to elucidate the complex structure of food toxins.^58^ Similarly, near infrared (NIR) spectroscopy uses NIR (14000–4000 cm^–1^) wavelength that causes vibration of C–H, O–H, N–H, and C=O bonds in the biomolecules and can be helpful for the detection of toxins in the food. Recently, this technique was used to detect Diarrhetic shellfish toxins in the mussels.^59^

### 2.4 Biological Assays

One of the most conventional methods is the direct injection of toxic samples into live animals and monitoring of their physiological response, behavior, and mortality. This assay is commonly used for the detection of marine toxins like Diarrhetic shellfish toxins in seafood.^60,61^ However, these tests are time-consuming and costly and raise major ethical concerns.

### 2.5 Biochemical Assays

Biochemical assays do not detect food toxins by direct measurement but rather involve the measurement of toxin-induced biochemical changes such as enzymes. For example, the inhibition of phosphatase activity is used to detect the presence of diarrhetic shellfish toxins.^62,63^ The use of this technique is very narrow, utilizing different instruments and reagents, and cannot be applied to multiple types of toxins, thus making it less applicable for the detection of various food toxins.

### 2.6 Microbiological Assays

Some food toxins are produced by certain microbes. Hence, assays involving the presence of the microbe are sometimes used as a proxy to detect their presence. For instance, the detection of *Bacillus cereus* in the food samples can point toward the presence of enterotoxins.^64,65^ Although these assays are easy to operate and inexpensive, they lack sensitivity and specificity.

### 2.7 Sensor-Based Approaches

Sensor-based methods of toxin detection in foods are very popular since they can be used on the site. There are many toxins such as aflatoxin B1, diarrhetic shellfish toxins, and microcystins that can be detected by sensors.^66^ Examples may be biosensors and aptasensors. Undoubtedly, the use of sensors helps to screen samples for the possible presence of toxins. However, the sensors may vary in terms of sensitivity and specificity.

## 3 Mass Spectrometry-Based Detection

Mass spectrometry (MS) is a powerful analytical technique used to detect and quantify various compounds including food toxins. This method can provide highly sensitive and specific results, making it a valuable tool for food safety and quality control.^67^ Infectious toxins like prions and Shiga toxins can also be analyzed using mass spectrometry, where peptides are digested by proteases and then the digested proteinaceous parts are analyzed by MS.^68^ The pervasive contamination of food products with mycotoxins has made monitoring their levels essential. Detection of mycotoxin biomarkers in urine provides valuable and specific data for exposure assessment to these food contaminants in order to overcome the disadvantages of the indirect approach based on food analysis.^69^ Due to the diverse chemistry and occurrence of food toxins in feedstuffs and foods with complex matrices, the detection has become difficult. The primary source of error in the analysis results from inadequate sampling and inefficient extraction and cleaning procedures. Gas chromatography (GC)-MS is used to analyze volatile and semivolatile compounds, such as certain mycotoxins and pesticide residues, in a variety of dietary products. Before entering the mass spectrometer for ionization and detection, the compounds are vaporized and separated according to their volatility.^70^ The principle of detection of food toxins using GC-MS includes multiple target analyte extraction using multiresidue analytical methods like QuEChERs and adsorption extraction.^71^

Recently, mass spectrometry has become one of the most effective methods even for identifying specific microorganisms by using matrix-assisted laser-desorption time-of-flight (MALDI-TOF) MS, followed by recognition of MS spectra unique to that organism that create a reliable fingerprint.^72^ MALDI-TOF MS-based identification of bacteria is more rapid, accurate, and cost-efficient than conventional phenotypic techniques and molecular methods. Rapid and reliable identification of food-associated bacteria is of crucial significance for product quality. In contrast to genotyping methods, it can also be readily implemented in routine analysis. Due to short turnaround periods, low sample volume requirements, and low reagent costs, MALDI-TOF MS has recently emerged as a powerful tool for the identification of food toxins or toxin-producing microorganisms.^73^ Food toxins from various fields of seafood, fruits, vegetables, milk, dairy products, and oils can be detected using MALDI-TOF MS.^74^ The microbial databases with unique features relevant to each microbial species are the key components and are therefore continually building up in size with the updated information on newly discovered microbial species and their annotations.

Another powerful MS-based tool routinely used for food toxin detection is liquid chromatography (LC)-MS due to its advantages in terms of sensitivity and selectivity. LC–MS is widely used for the analysis of mycotoxins, alkaloids, marine toxins, glycoalkaloids, cyanogenic glycosides, and furocoumarins in food. The excellent sensitivity, even at low concentration levels, selectivity, and capacity to resolve coeluting compounds based on their molecular masses make LC–MS currently the most effective technique for the simultaneous detection of multiple regulated, unregulated, and emerging toxins in a single run. Commonly, the LC–MS methods for the quantitative determination of natural toxins are based on the use of a triple-quadrupole analyzer, tandem mass spectrometry, and multiple reaction monitoring (MRM) modes. The co-occurrence of natural toxins in combination with other chemical contaminants, such as pesticides, growth regulators, and veterinary drugs, as well as bioactive compounds (i.e., lignans, flavonoids, and phenolic compounds), in a wide variety of food matrices has increased the demand for analytical methods addressing the simultaneous determination of multiple analyte classes. LC–MS method used for the detection of food toxins includes alkaloids, furocoumarins, cyanogenic glycosides, marine toxins, and mycotoxins.^41^

Inductively coupled plasma mass spectrometry (ICP-MS) is a powerful analytical technique for the detection of elementals like heavy metals that allow multielement detection simultaneously with high speed and at very low concentrations.^75^ Further, for the enhanced selectivity, inductively coupled plasma mass spectrometry (ICP-MS) and tandem mass spectrometry (MS/MS) can be utilized. In the ICP-MS technique, the sample under examination is digested by employing a suitable technique such as dry ashing, acid digestion, or microwave digestion, etc. to solubilize the analytes of interest. Further, the sample is injected into an inductively coupled plasma source which ionizes the sample and detected by MS.^76^ Ion mobility spectrometry (IMS) is another advancement in analytical techniques that relies on ion mobility that is under the influence of velocity of ions and strength electric field.^77,78^ In order to take advantage, multiple MS-based approaches can be merged and operated together, which improves the efficiency of analytical techniques. Tandem mass spectrometry (TANDEM MS), also called MS/MS, is one such approach in which samples are analyzed either by multiple mass spectrometers connected to each other or with different analyzers arranged sequentially.^79^ A MS technique can also be coupled with immunoaffinity chromatography, and it is known as IAC-MS. This technique uses antibodies-based columns to acquire selectivity and also to isolate target analytes from the sample matrix.^80^ The isolated target molecules are injected into the MS component, which provides high sensitivity and identification of toxins based on mass-to-charge ratio. IAC-MS provides exceptional selectivity and can be used for the detection of various types of food toxins such as mycotoxins, pesticides, veterinary residues, and allergens, etc.^81^ MS-based approaches have offered diverse and advanced modules for the detection of analytes precisely. Table 2 summarizes the advantages and disadvantages of MS-based approaches under different conditions.

**Table 2:** Variants of Mass Spectrometry (MS) and Their Advantages and Disadvantages

Technique(s)	Important features	Advantages	Disadvantages	Ref
Accelerator mass spectrometry (AMS)	• Employed particle accelerator technology into a mass spectrometer.	• Small quantity of sample is sufficient	• High cost makes it less affordable	(82, 83)
	• Its detection range include ion currents of more abundant stable isotopes (e.g., 12C, 13C) to very rare radionuclides (e.g., 14C)	• Need less time for estimation	• Small sample size makes it prone to contamination	
Liquid chromatography mass spectrometry	• Simple and robust technique for regular analysis	• Wide linear dynamic range	• Lower accuracy	(84, 85)
	• Able to detect nonvolatile compounds like sugar and proteins that cannot be detected in GC-MS	• Lower detection limit	• Isotopes cannot be detected	
		• High precision and accuracy		
Gas chromatography mass spectrometry	• Sample exposed to high temperature	• Analysis is faster and selectivity,	• Destructive method of analysis	(86−88)
	• Used for the detection of volatile compounds from sample	• Lower detection limits	• Only thermolabile compounds can be detected	
	• Nonvolatile compounds like sugar can be detected after dertivatization			
High resolution mass spectrometry	• Good for the identification of unknown samples	• Highly accurate and selective measurement	• Expensive analysis	(89)
	• Efficient for nontargeted analyses	• Able to detect mass accurately with even small change is detectable	• Data generated is hige and complex	
			• Not suitable for regular analysis of known samples	
Matrix-assisted laser desorption-ionization time-of-flight mass spectrometry	• Traditional method for the identification of microorganisms	• Able to differentiate between phenotypic, genotypic, and biochemical properties	• In some cases, unable to differentiate between closely related species, e.g., *E. coli*, and *Shigella*	(90)
	• Sample is first ionized, and segregated based on mass-to-charge ratio	• Reduced analysis time	• Lack sufficient spectra in database	
	• Measurement is done by determining with time-of-flight			
Inductively coupled plasma mass spectrometry	• Used to measure the element level in sample	• Wide analytical range with lower detection limit	• High cost of investment and operation	(91)
	• Sample converted to aerosol from liquid	• Need small quantity of sample	• Need experts for operation	
		• High throughput with multielement detection		
Surface-enhanced laser desorption/ionization time-of-flight mass spectrometry	• It is also known as SELDI-TOF-MS-based ProteinChip System	• It employes chromatographic separation techniques	• Low detection precision for individual proteins from complex	(92, 93)
	• It is modified form of MALDI-TOF	• Less time-consuming and high through put system	• Low mass resolution	
	• Proteomic profiling of biological fluids			
Tandem mass spectrometry	• Two or more MS units are interconnected with quadrupoles and TOF analyzer	• Highly specific	• High operational cost	(79, 94, 95)
	• Effective in analyzing complex mixture	• Low signal-to-noise ratio	• Limited sample through put	
		• Able to detect covalent modifications in proteins		
		• Sensitive and reproducible		

## 4 Recent Advancements in Food Toxin Detection Using MS

Food toxins have become a serious concern for the society. The presence of various types of contamination and toxins like pesticides, herbicides, microbial metabolites, and plant-based toxins is highly detrimental to human health even at ppb concentrations when present in food, water, or animal feed.^96^ Safe food is explicitly a matter of concern and is indispensable for human health. In the current scenario, an effective and sensitive detection method becomes necessary to detect contamination of food and water with chemicals and pathogenic microbes and related products. Existing conventional methods for food analysis, based on PCR, chromatography, and spectrophotometry, have shown significant reliability and accuracy; however, the cost of analysis, time consumption, and requirement of specialized personnel impede their usage for frequent monitoring of food samples (as already summarized above). Hence, there exists an upsurging thrust for innovating rapid, accurate, robust, but inexpensive alternatives for in situ and real-time detection of contamination of food samples. The primary step involved in every methodology to identify the food toxins in the sample under examination involves the extraction of food toxins from the matrix followed by purification to remove other substances that can interfere with the analysis.^97^ After successful extraction of toxins, the sample is ionized into ions with ionization techniques such as electrospray ionization (ESI), chemical ionization (CI), or APCI (atmospheric pressure chemical ionization) or desorption techniques such as matrix-assisted laser desorption ionization (MALDI). These ions from the ionization chamber are accelerated followed by deflection in the magnetic field due to a difference in their masses. The beam of ions is then analyzed by the detector based on their mass-to-charge ratio. There are many types of mass analyzers available such as magnetic sector analyzers, quadrupole mass analyzers, double focusing analyzers, time-of-flight analyzers, etc. Further, MS can also distinguish between different food toxins depending upon their mass-to-charge ratio. In order to overcome the limitations associated with the conventional methods, MS can be coupled with these techniques to enhance the capabilities in complete and accurate analysis of different food toxins present in the sample.^98^ GC technique relies on the comparison of retention times with known standards and also lacks in the distinction of structurally similar compounds. Thus, to enhance selectivity, identification, and elucidation of the structure of various toxins present in the sample under examination, GC is coupled with the MS.^99^ HPLC technique requires optimization such as the selection of columns and mobile phases for each specific class of toxins. In addition to this, the sensitivity of HPLC is also low as that of MS techniques. HPLC techniques are more time-consuming than MS as they involve complex procedures for sample preparation.^100^ We are herein summarizing various types of food toxins and the recent advancements in their detection using MS.

### 4.1 Mycotoxins

Mycotoxins are toxic secondary metabolites produced by fungi. These toxins can accumulate in the fungal-contaminated food grains such as corn, cereals, and legumes, and upon ingestion can traverse into the food chain affecting humans and animals.^101^ As per the Rapid Alert System for Food and Feed of EU (RASFF) report, mycotoxins contaminate around one-quarter of global food grain production both during pre- and postharvest.^102^ This clearly indicates the severity of the mycotoxin problem in the food that we consume. In the literature, around 300 mycotoxins are reported, but only seven toxins are quite common in food worldwide such as aflatoxins (AF), trichothecenes (TC), zearalenone (ZEN), fumonisins (FB), ochratoxins (OTA), citrinin (CIT), and patulin (PAT).^101^ Fungal species belonging to the genera of *Aspergillus*, *Penicillium*, and *Fusarium* are predominantly toxigenic and most frequently lead to cases of mycotoxin concentration.^101^ AF (B1, B2, G1, and G2 produced by *Aspergillus*), CIT (by *Penicillium*, *Aspergillus*, and *Monascus* etc.), ergot alkaloids (*Claviceps purpurea*), FB (*Fusarium* sp.), ochratoxin A (produced by *Penicillium* and *Aspergillus*), PAT (*Penicillium patulum*), TC (*Fusarium*, *Stachybotrys*, *Trichothecium*, and *Trichoderma* sp.), and ZEN (*Fusarium graminearum*) are some of the well-known mycotoxins responsible for serious lethal reactions like cancer induction, kidney toxicity, immune suppression, stachybotryotoxicosis, turkey X syndrome, etc.^103^ Another issue with these mycotoxins is their resistance and tolerance toward thermal treatment; hence, they remain active even after heating.^104^

**Figure 2:**
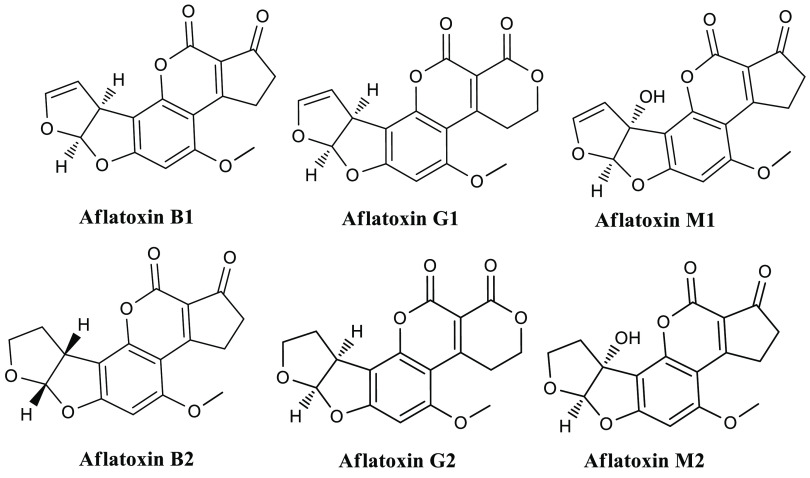
General structures of some of the Aflatoxin variants.

AFs are groups of potentially toxic fungal secondary metabolites reported from *Aspergillus flavus*, *A. parasiticus*, and *A. nomius* (Figure 2). They are produced from polyketides and commonly present in cereal crops like corn, peanuts, walnuts, wheat, etc.^105^ AFs can lead to chronic toxicity related to hepatic tissues, necrosis, hepatomas, periportal fibrosis, jaundice, hemorrhage, and fatty liver changes, and also exhibit teratogenicity, carcinogenicity, and immunotoxicity.^105,106^ They were first reported in the state of Gujrat and Rajasthan, India in 1974, which resulted in the onset of hepatitis caused by *A. flavus* infected staple food and maize. *A. flavus*, *A. parasiticus*, *A. nomius*, and sometimes *Emericella* spp. are the main producers of AFs.^105^ To date, more than 20 AFs variants have been reported, among which variant B1 is the most common and most lethal. AFs B1 and B2 are produced by *A. flavus* and *A. parasiticus*, and AF M1 is produced by *A. parasiticus* and can be transmitted through milk. Variants M1 and M2 are also produced by metabolism of B and B2.^16^ AF B1 is metabolized by the P450 monooxygenase system and generates AF 8,9-epoxide (reactive epoxide) that induces mutations and cancer by forming DNA abducts.^106^

**Figure 3:**
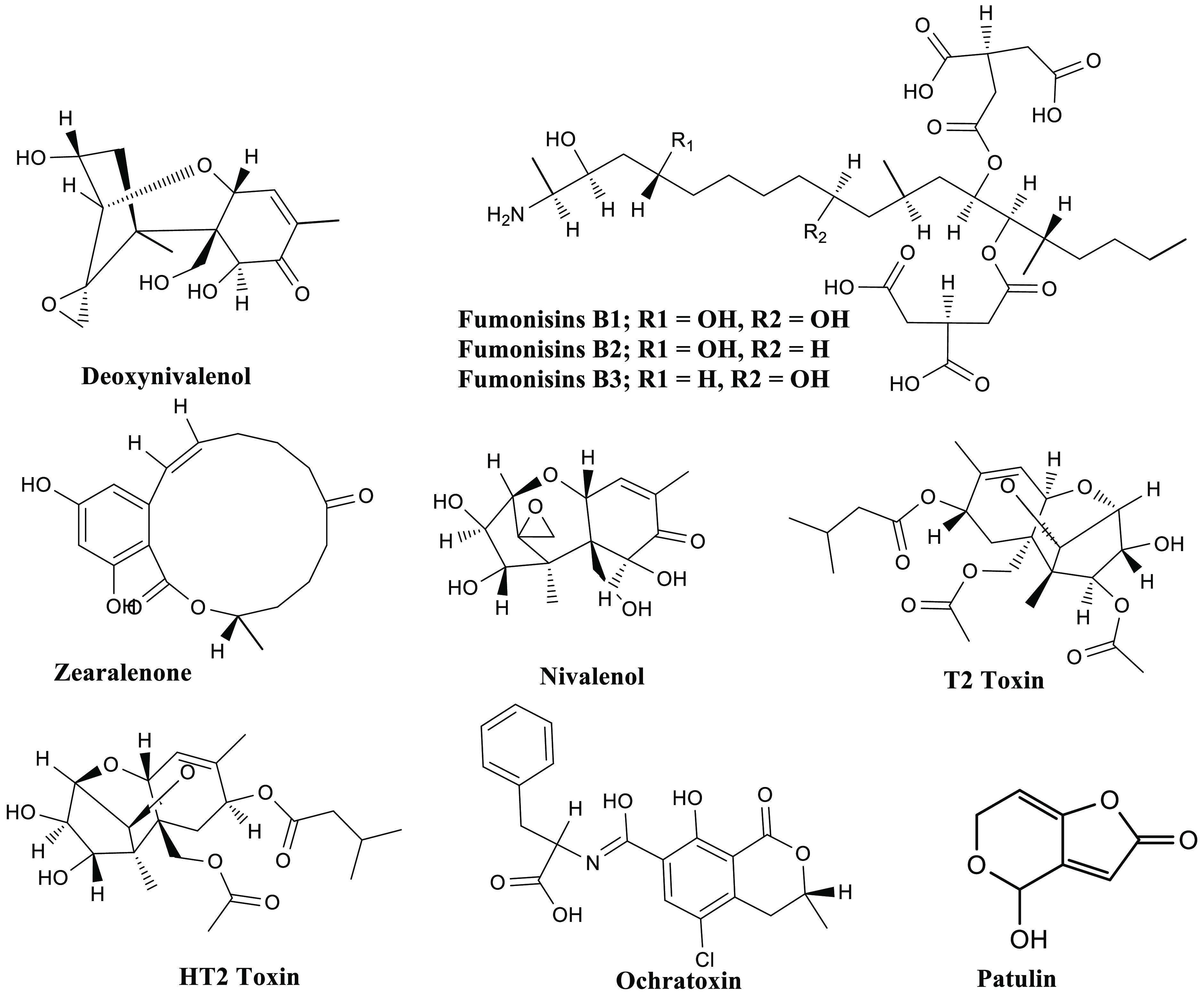
General structures of various mycotoxins.

Besides *Aspergillus*, isolates belonging to *Fusarium* are also known to produce the most potent toxins, which include deoxynivalenol (DON), FBs, and ZENs (Figure 3). DON belongs to the sesquiterpenoid group of trichothecenes. It mainly contaminates corn, wheat, and barley. It is also a toxic secondary metabolite that has a negative health impact on the consumer by disturbing the intestinal barrier and has immune-stimulatory as well as immune-suppression properties at low and high doses, respectively.^107^ The same group also contains its acetylated derivatives named nivalenol, T-2 toxin, and HT-2 toxins.^108^ After ingestion, DON is absorbed and metabolized in the intestine via DON-3S, DON-GlcA, and DOM-1. In poultry birds, DON-3S and DON-15S are eliminated via bile and urine, while in swine, it is absorbed in the upper digestive system; hence, poultry birds are the least sensitive to these toxins, and swine are most sensitive to these toxins. In humans, contaminated foods like infected meat and cereals are the most common sources.^107^*Fusarium* sp. is also responsible for other mycotoxins named FBs (secreted by *Fusarium verticillioides* and *Fusarium proliferatum*). *Aspergillus niger* is also able to produce FBs. Such toxins are commonly reported from cereals like peanuts, maize, rye, oats, millets, and grape.^109^ To date, more than 15 homologous forms of fumonisin have been reported that are referred to as A, B1, B2, B3, C, P, etc. However, B1 is the most toxic form of FB.^110,111^ FBs are known to have carcinogenic, neurotoxic, and hepatoxic effects that cause hepatocarcinoma, defects in the neural-tube, and nephrotoxicity.^109^ ZEN is a nonsteroidal, estrogenic mycotoxin produced by *F. acuminatum*, *F. crookwellense*, *F. culmorum*, *F. cerealis*, *F. equiseti*, *F. graminearum*, *F. oxysporum*, *F. sporotrichioides*, *F. semitectum*, and *F. verticillioides*.^112^ It disrupts reproductive capacity by affecting mammalian folliculogenesis and impairs granulosa cell development and follicle steroidogenesis.^113^ It is thermostable^114^ and resistant to processing stress like milling and storage.^115,116^ ZEN leads to kidney damage and liver injury and causes inflammation.^112^

Ergot alkaloids are toxic secondary metabolites produced by several fungi of *Clavicipitaceae* (*Epichloë*, *Claviceps*, *Balansia*, and *Periglandula*) and *Aspergillus fumigatus*. The producers mainly include *Epichloë endophytes*, *Epichloë festucae var. lolii*, *Epichloë coenophialum*, and *Claviceps purpurea*.^117^ They belong to compounds containing an indole group and are derived from L-tryptophan. They cause “ergotism” and their toxic effect is reflected by hyperglycemia, gastrointestinal upset, mydriasis,^118^ and even endocrine disruption.^119^ Besides these major mycotoxins, some other chemicals including OTA and PAT have also been reported from fungi. OTA is nephrotoxic and produced by a diverse range of fungi such as *Aspergillus ochraceus*, *A. carbonarius*, *A. niger*, and *Penicillium verrucosum*. It can also lead to renal tumors, Balkan endemic nephropathy, and chronic interstitial nephropathy.^120^ Patulin is produced by *Penicillium*, *Aspergillus*, and *Byssochlamys*, while alternariol (AOH) and alternariol monomethyl ether (AME) are *Alternaria* toxins, produced by fungi of the *Alternaria* genus found in fruits and related products.^20^

To ensure food safety with special consideration to mycotoxins, international, national, and regional agencies like the World Health Organization, Food Agriculture Organization, Codex Alimentarius Joint Expert Committee for Food Additives and Contaminants, European Food Safety Authority, GCC Standardization Organization, and Japanese Association of Mycotoxicology have determined the permission limit for mycotoxins contamination in food as well as feed.^121^Table 3 summarizes the permissible limit for various mycotoxins in food^122−125^ and animal feed^126,127^ as per the European Commission.

**Table 3:** Regulatory Permission Limits for Various Mycotoxins in Food and Feed Products As Per European Commission^122−127^

Category	Food or animal feed products	Permissible limit (μg/kg)
Aflatoxins B1 (AFB1)		
Food	Brazil nuts, groundnuts, hazelnuts, and oilseeds for human consumption after physical treatment	8
	Almonds, apricot kernels, and pistachios for human consumption after physical treatment	12
	Brazil nuts, groundnuts, hazelnuts, and oilseeds for human consumption directly (No physical treatment)	2–5
	Almonds, apricot kernels, and pistachios for human consumption directly (No physical treatment)	8
	Dairy products for consumption by infants, baby food, and processed cereal-based food	0.1
	Spices	5
	Dried fruits, and Figures for human consumption after physical treatment	5–6
	Dried fruits (except Figures) for human consumption directly (No physical treatment)	2
	Maize and rice (as ingredients) for human consumption after physical treatment	5
Feed	Feed materials	0.02–0.05
	Complete feeding stuff with the exception of	0.05
	Calves, cattle, and lambs	0.005–0.01
	Poultry	0.02
	Complementary feeding stuff	0.005–0.05
Aflatoxins M1 (AFM1)		
Food	Milk	0.05
	Infants’ dairy products, baby formula and baby milk,	0.025
Aflatoxins (AFs) total		
Food	Almonds, apricot kernels, brazil nuts, groundnuts, hazelnuts, oilseeds, and pistachios for human consumption after physical treatment	15
	Groundnuts, oilseeds, and processed products for human consumption directly (No physical treatment) or as ingredient	4
	Almonds, apricot kernels, brazil nuts, hazelnuts, pistachios, and for human consumption directly (No physical treatment)	10
	Spices	10
	Dried fruits, and Figures for human consumption after physical treatment	10
	Dried fruits (except Figures) for human consumption directly (No physical treatment)	4
	Maize and rice (direct or as ingredients) for human consumption after physical treatment	10
Citrinin (CIT)		
Food	Food supplements prepared from red yeast fermented rice	2000
Deoxynivalenol (DON)		
Food	Unprocessed durum, maize, oats and wheat	1750
	Cereals and cereal flour for direct human consumption	750
	Cereal-based processed foods and baby food	200
Feed	Animal feed from–cereals	8–10
	Complete as well as complementary feeding stuff	0.9–5
Fumonisin (FB1+FB2)		
Food	Unprocessed maize	4000
	Maize for direct human consumption	1000
	Maize-based processed food for babies and young children	200
Feed	Maze based feed	60
	Complete and complementary feeding stuff	5–50
Ochratoxins (OTA)		
Food	Cereals-based unprocessed products	3–5
	Cereal-based processed food and baby food, dietary foods products specially for medical purposes purposes	0.5
	Beverages based on grapes	2
	Coffee roasted/instant	5/10
	Spices	15
Feed	Cereals-based feed materials	0.25
	Complete and complementary feeding stuff for poultry	0.1
Patulin (PAT)		
Food	Fruit juices	50
	Solid apple products	25
	Solid apple as well as apple juice for babies and young children	10
Zearalenone (ZEN)		
Food	Cereal products (unprocessed except maize)	100 (350)
	Cereals for direct human consumption	75
	Maize for direct human consumption	100
	Cereals and maize processed products for babies and young children	20
Feed	Cereals and maze-based feed materials	2–3

HPLC and GC are the common approaches for the detection and higher accuracy and sensitivity making MS an elegant and dominant analytical tool for toxicological and metabolite analysis.^128^ In addition, it also allows simultaneous detection of a diverse range of toxins together and aids in method standardization and implication to ensure the rapid evaluation of samples for food safety analysis. Areo et al.^129^ have employed UHPLC–MS/MS for the detection of AFs, ZEN, and OCT A from 100 tea samples (collected from registered shops within South Africa), prepared in acetonitrile/water/acetic acid solvent by QuEChERS extraction method. The supernatant was mixed with 900 mg of anhydrous MgSO_4_, 150 mg of C18, and 150 mg of primary secondary amine that separates the organic phase. The organic phase was collected and dried under a nitrogen stream and further reconstituted in methanol/water for analysis via UHPLC–MS/MS. The method selected has very high linearity (>0.99) and precision (6–29%). AFs, i.e., AFB1, AFB2, AFG2, OCT A, and ZEN, were absent in the samples, and AFG1 was present in very low amounts 1.72–5.19 μg/kg that were also below the regulated level in food as recommended by EU Commission Regulation 1881/2006.^129^ You et al.^130^ have evaluated the effect of culture medium for mycotoxin accumulation by *Alternaria*. Secondary metabolites were characterized by nontarget analysis with HRMS. Mycotoxins produced by *Alternaria* were grouped into for families: alternariol monomethyl ether (AME), alternariol (AOH), altenuene (ALU), Desmethyl dehydro altenusin (DMDA), and dehydroaltenusin (DHA) families, Altertoxin-I (ATX-I) family, tentoxin (TEN) family, and tenuazonic acid (TeA) family. Culture medium greatly influenced the type of mycotoxins produced. *wiz* Potato Sucrose Agar medium is suitable for AOH, AME, ALU, ALT, DHA, and DMDA, while Potato Dextrose Agar supported the accumulation of ATX-I, TEN, and TeA. Table 4 summarizes some of the research for the detection of various mycotoxins with MS-based tools.

**Table 4:** Detection of Mycotoxins by MS-Based Approach

Name	Sample	Method	Operating parameters	Outcome	Ref
Trichothecenes T2	Wheat and maize	Portable mass spectrophotometer	MS parameter: heater 200 °C; sample pump 0%; mass center: *m*/*z* = 484; mass tolerance: 1; spectrum average: 10	Limit of detection 0.2 mg/kg	(131)
*Alternaria* toxin	Fruits and vegetables	QuEChERS extraction followed by MS analysis	Triple quadrupole MS; pressure curtain gas 35 psi and collision gas 8 psi; ion spray voltage 5,500 V (positive ion mode), and −4,500 V (negative ion mode)	Detection limit 1.0–5.0 μg/kg (Extraction recoveries 73.0–120%)	(132)
				Repeatability <12.9%	
Mycotoxins	Standard sample	UHPLC-QTrap-MS/MS	Injection volume: 20 μL; column temperature: 30 °C; flow rate: 0.2 mL min^–1^; mobile-phase 0.1% formic acid+ acetonitrile run time: 25 min	Limit of quantification 0.005 and 13.54 ng mL^–1^ (Limit of detection 0.001–9.88 ng mL^–1^; 0.005; Recovery 67.5–119.8%)	(133)
Aflatoxin B1 and M1	Blood	HPLC–MS/MS	Allure PFPP column; positive electrospray ionization; source temp 500 °C	LOD 0.05 to 0.2 ng/mL; accuracy 92–111%	(134)
Alternaria toxins	Vegetable sample	UPLC–MS/MS	ACQUITY UPLC BEH C18 column; injection volume: 5 μL, column temperature: 40 °C; flow rate 0.4 mL min^–1^, mobile phase: 0.1% formic acid water; gradient flow 10–90%	Contaminated solanaceous vegetable: 41.1%	(135)
				AME: 4.26%; AOH: 6.38%; altenuene: 6.38%; tentoxin: 42.6%; tenuazonic acid: 55.3%	
Aflatoxin M1		HPLC–MS/MS	Quantitative daughter *m*/*z*: 273; qualitative daughter *m*/*z*: 259; collision energy 23 eV	Quantification limit 1.62 ppb; detection limit 0.54 ppb; 98.5% accuracy	(136)
Ochratoxin A	Coffee and tea	UHPLC–MS/MS	Triple quadrupole MS; electrospray ionization, ion source temp 300 °C; flow rate 3 L/min	Sensitivity 0.30 and 0.29 ng/mL	(137)
Trichothecenes	Oat based products	U-HPLC–HRMS/MS	Reverse phase column; electro-ionization detector; column temperature 40 °C; ethanol+water+formic acid gradient elution	Frequency of free T2 toxin 92%	(138)
				T2 mono glucoside 69%	
Alternaria toxins	Rice	LC–MS/MS	Separation with hyper clone column and detection with C18 column; detection with negative electrospray ionization, source temperature 300 °C	Limit of detection and quantification	(139)
				AME: 0.03 and 0.09 μg/L; altenuene: 5.48 and 16.24 μg/L	
Alternariol monomethyl ether (AME), Alternariol (AOH), and tentoxin	Standard sample	LC-ESI-MS/MS	LC–MS/MS with triple quadrupole; separation at 25 °C with C18 column; analysis with quadrupole MS, source temperature 450 °C; ion spray voltage 5500 V	Limits of detection and quantitation: 0.7 and 3.5 ng/g recovery 80%	(140)

### 4.2 Bacterial Toxins

Bacterial contamination has shown diverse causes as *Shigella* mainly infect via unwashed hands, while *Campylobacter* and *Escherichia coli* are usually present in raw milk, undercooked meat and poultry products, and contaminated water. In contrast, *Listeria monocytogenes* and *Yersinia enterocolitica* are found in refrigerated food.^141^ Bacterial toxins have been classified as endotoxins and exotoxins. Structurally, endotoxins have distinct structural regions, i.e., glycolipid is made up of disaccharide and fatty acids which are usually capric, lauric, myristic, palmitic, and stearic acids. These acids are buried within the outer cell membrane of the bacterium. The nucleus is the second important part, which is made up of a hexose- and heptose-based heteropolysaccharide. The glycolipid and nucleus are interconnected by the sugar acid 2-keto-3-deoxyoctanate. Endotoxins are lipopolysaccharides and are part of the outer membrane of Gram-negative bacteria. These are also identified as important determinants and antigenic parts of bacteria that aid in attachment with the host as well as in pathogenicity. Exotoxins are proteins in nature that are released by Gram-negative bacteria and disrupt cell division, causing lysis and tissue damage.^142,143^ Exotoxins are further classified into types I, II, and III based on the mechanism of action. Toxin type I can make critical changes in the host’s cells without internalizing. Superantigens secreted by *Staphylococcus aureus* and *Streptococcus pyogenes* are examples of a type I toxin. The type II group includes hemolysins, phospholipases, aerolysin, and GCAT proteins. It intrudes the host cells and creates pores to destroy the host cell’s membrane. In comparison to type I and II, type III is a diverse group in terms of activity. It has a binary structure with fractions A and B. Fraction B in the toxin facilitates the binding with receptor in the host cell, while another fraction, i.e., “A” carries enzymatic activity and is responsible for the toxin effect. Anthrax toxin (*Bacillus anthracis*), Cholera toxin (*Vibrio cholerae*), and Shiga toxin (*Escherichia coli* O157:H7) are some examples of exotoxins.^142,144^ Botulinum is 150 kDa and composed of a heavy chain of 100 kDa and a light chain of 50 kDa. Heavy chain is responsible for binding to receptors on neuron surface, while light chain cleaves proteins required for nerve signal transmission, i.e., botulinum A, C, and E cleave synaptosomal-associated protein 25, while B, D, F, and G variants act on synaptobrevin-2.^72,145,146^ In a similar fashion, *B. anthracis* produces three types of proteins or factors, e.g., lethal factor, edema factor, and protective antigen. Protective antigens split and form a fragment of 63 kDa that forms heptamers and octamers to finally find the cell surface. In addition, they also bind with lethal factors to form lethal toxin.^72,147^

#### 4.2.1 Detection of Bacterial Food Toxins

*Clostridium*, *Salmonella*, *Staphylococcus*, and *Listeria* are some common pathogens causing foodborne infections in humans. Previously used methods, e.g., enzyme immunoassay (EIA), were fast and sensitive, but their accuracy was limited due to cross-reactivity reporting a high rate of false positives and misguided public health care personnel. Techniques based on MS are powerful and can be multiplexed for the detection of various protein toxins, e.g., toxins from *Clostridium*,^148^*Bacillus*,^88^ and many more with speed, sensitivity, and accuracy. Botulinum, a neurotoxin, is produced by *Clostridium botulinum* in seven different serotypes (A–G). Specific detection of these toxins from different strains needs high analytical sensitivity and a MS-based approach. The enzymatic activity-based approach relies upon substrate fragments generated by these toxins which can be used as targets by MALDI-TOF MS.^72^

In 2002, a peptide mass map of toxin variants A1 and B1 was prepared by targeting the trypsin digest of the toxin by van Baar and colleagues. The work was extended to C, D, E, and F in 2004.^72^ In successive generations, several advancements have shown effective approaches like endopep-MS.^149^ Rosen et al. have developed the endopep-MS-based method for the identification of botulinum A and E simultaneously and rapidly.^150^ Both A and E identify the same target SNAP 25 protein but act on different sites. 3D structures of both types of fragments were used for differential identification. Drigo et al.^151^ employed the same endoPep-MS approach for botulinum toxins C and D. The method has shown a sensitivity of 100% with specificity and accuracy of 96.08% and 97.47%, respectively. Integration of MS with other analytical methods like HPLC, GC, FPLC, etc. has improved bacterial toxin profiling. Toxoflavin and fervenulin are bacterial toxins produced by *Berkholderia* and *Streptomyces hiroshimensis*. These compounds are common contaminants in fermented corn flour, rice bran oil, distiller’s yeast, sweet potato starch, *Tremella fuciformis* Berk., and rice noodles. These compounds are sensitive to degradation in 1% ammonia solution. UHPLC-Q-TOF/MS allowed for the detection of degradation products. The modified approach led to lower down the limits of detection of toxoflavin and fervenulin to 12 μg/kg and 24 μg/kg, respectively, with recovery of 70.1–108.7%.^152^ The MS approach also employed natural phenomena of antigen–antibody interactions for the detection of toxins and related antigens. *Salmonella typhi*, Gram-negative enterobacteria, is responsible for typhoid fever and meningitis. The immunoreactive proteins of bacteria were used as targets to develop improved diagnostic tools with MS. An immunoaffinity-based proteomic approach was employed with IgG and IgM antibodies from typhoid patients. The approach aided in the identification of 28 immunoreactive proteins, out of which 14 were complementary to IgG, 4 for IgM, and the rest 10 for both, hence retained by respective charged columns. In context to antigenicity, 22 proteins have shown antigenicity and immunogenicity.^153^ Such an approach is helpful for rapid identification, and its reproducibility and reliability can be employed for vaccine and drug development. Peptide mass fingerprinting technique (PMF) associated with MALDI TOF/MS or ESI/MS is a top-down MS protocol where proteins are directly ionized to create a fingerprint of individual proteins and applied for detection of various microbial strains. PMF of unknown organisms is compared to those existing in PMF databases or compared to the spectrum of biomarker proteins with the proteomic spectral database, using MALDI-TOF MS. Typically a mass range *m*/*z* of 2–20 kDa is used for species-level identification where ribosomal proteins representing 60–70% of microbial cells’ dry weight and some housekeeping proteins are selected.^154^ Thus, by comparing with extensive commercial databases, microbial contaminants can be traced to the genus, species, or strain level, and such an identification tool is conveniently adapted in diagnostic laboratories.^155^ However, using biomarkers is not very common for identification since it requires prior insight into the genome sequences before creating the required databases for proteins’ molecular masses. *Staphylococcus aureus* delta-toxin has been detected using whole-cell (WC) MALDI-TOF/MS and LC–MS to correlate the expression of delta-toxin with the status of the agr (accessory gene regulator) status. Mass spectra of pure toxin from wild type strains and mutants for agr-rnaIII gene were compared specifically at the position of the peak for delta-toxin.^156^

Biosensors have taken up an important role in the accurate and fast detection of food contaminants even in very low concentrations^157^ using biorecognition elements, such as antibodies, enzymes, nucleic acids, phages, etc., along with electrochemical, optic, or piezo-electric devices for the detection of food contaminants. MS-based biosensors are less prevalent as compared to optical or electrochemical ones^158,159^ but have the potential to overcome the drawbacks of conventional models of biosensors. A multitoxin biosensor-MS was developed for the detection of multiple bacterial toxins simultaneously. Biomolecular interaction analysis-MS (BIA-MS) that used a two-step method, i.e., first bonding of toxin molecules to antibodies immobilized on a sensor chip using SPR (surface plasmon resonance) and then the bound toxin, was identified by MALDI-TOF/MS. The potential of the multiaffinity sensor chip was validated by the detection of endotoxin from *Staphylococcus* in mushroom and milk samples and it successfully detected multiple toxins at concentrations as low as 1 ng/mL.^160^

### 4.3 Marine Biotoxins

**Figure 4:**
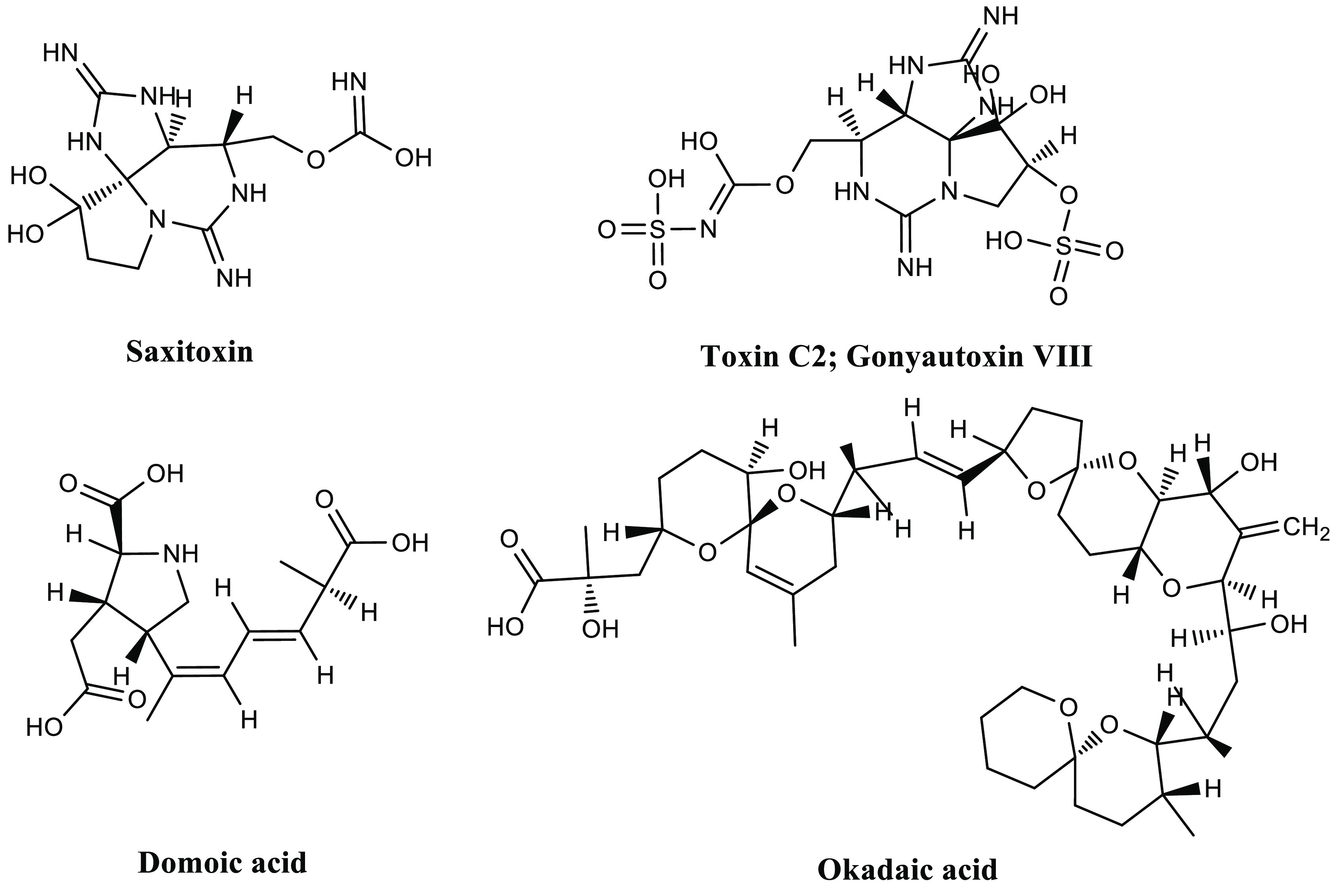
General structures of various marine biotoxins.

Marine biotoxins are natural compounds released in the marine environment by algae and phytoplankton during harmful algal blooms (Figure 4). These compounds are highly toxic for consumers and not only are related to serious illness but also lead to the death of aquatic organisms and even humans.^161^ Due to continuous release in the surrounding environment, these biotoxins accumulate in aquatic and marine organisms such as mollusks and fishes. Based on the chemical nature and solubility, these biotoxins are hydrophilic and lipophilic. Hydrophilic biotoxins are water-soluble and can cause amnesic shellfish poisoning, paralytic shellfish poisoning, and emerging pufferfish poisoning, while other groups of lipid-soluble biotoxins are responsible for diarrhetic shellfish poisoning and azaspiracid shellfish poisoning. There is another group of toxins with less available information that is categorized as emerging toxins and can cause unregulated ciguatera fish poisoning, cyclic imines, and neurotoxic shellfish poisoning.^162−165^ Paralytic shellfish poisoning (PST) is a group of more than fifty-eight related compounds, produced by *Alexandrium* dinoflagellates of the Atlantic and Pacific coast and Mediterranean Sea. It has a tetrahydropurine skeleton among which saxitoxin (SXT) and gonyautoxin (GNT) are common. Structurally, STX has been categorized into four subgroups named carbamate, N-sulfo-carbamoyl, decarbamoyl, and hydroxylated saxitoxins.^166^ The toxicity related to PST is reflected in mild as well as severe depending upon toxicity. The mild symptoms include numbness, tingling sensation around lips followed by expansion of the area, itching and prickly sensation in fingertips and toes, dizziness, headache, and nausea. Moderate and severe illness symptoms include incoherent speech, prickly sensation and stiffness in limbs, weakness, difficulty in respiration, and muscular paralysis.^161^

Amnesic shellfish poisoning is mainly caused by domoic acid (DA) and derivatives produced by marine diatoms of *Pseudonitzschia*. DA, cyclic tricarboxylic amino acid, can bind with glutamate receptors in the central nervous system due to structural analogy and result in excess stimulation, induced production of reactive oxygen species (ROS), and ultimately cell death.^167^ Consumption of DA resulted in gastrointestinal ailments including abdominal cramps, diarrhea, nausea, and vomiting. Neurological symptoms may also include confusion, disorientation, paresthesia, lethargy, short-term memory loss, and in severe toxicity cases, it may also result in coma or death.^168^

Diarrheic shellfish poisoning (DST) is a toxin produced by dinoflagellates of *Dinophysis* and *Prorocentrum.* It is a common type of contamination in shellfish industries due to overextended prohibitions on mussel harvesting activity.^169^ The responsible toxins for DST are a group of polyether compounds recognized as okadaic acid and its derivatives (dinophysistoxin); pectenotoxin; yessotoxin and its derivatives; and azaspiracid. Okadaic acid and azaspiracid consumption resulted in abdominal pain, diarrhea, nausea, and vomiting.^161,170^ Pectenotoxin and yessotoxin are not involved in human illness.^171^

**Figure 5:**
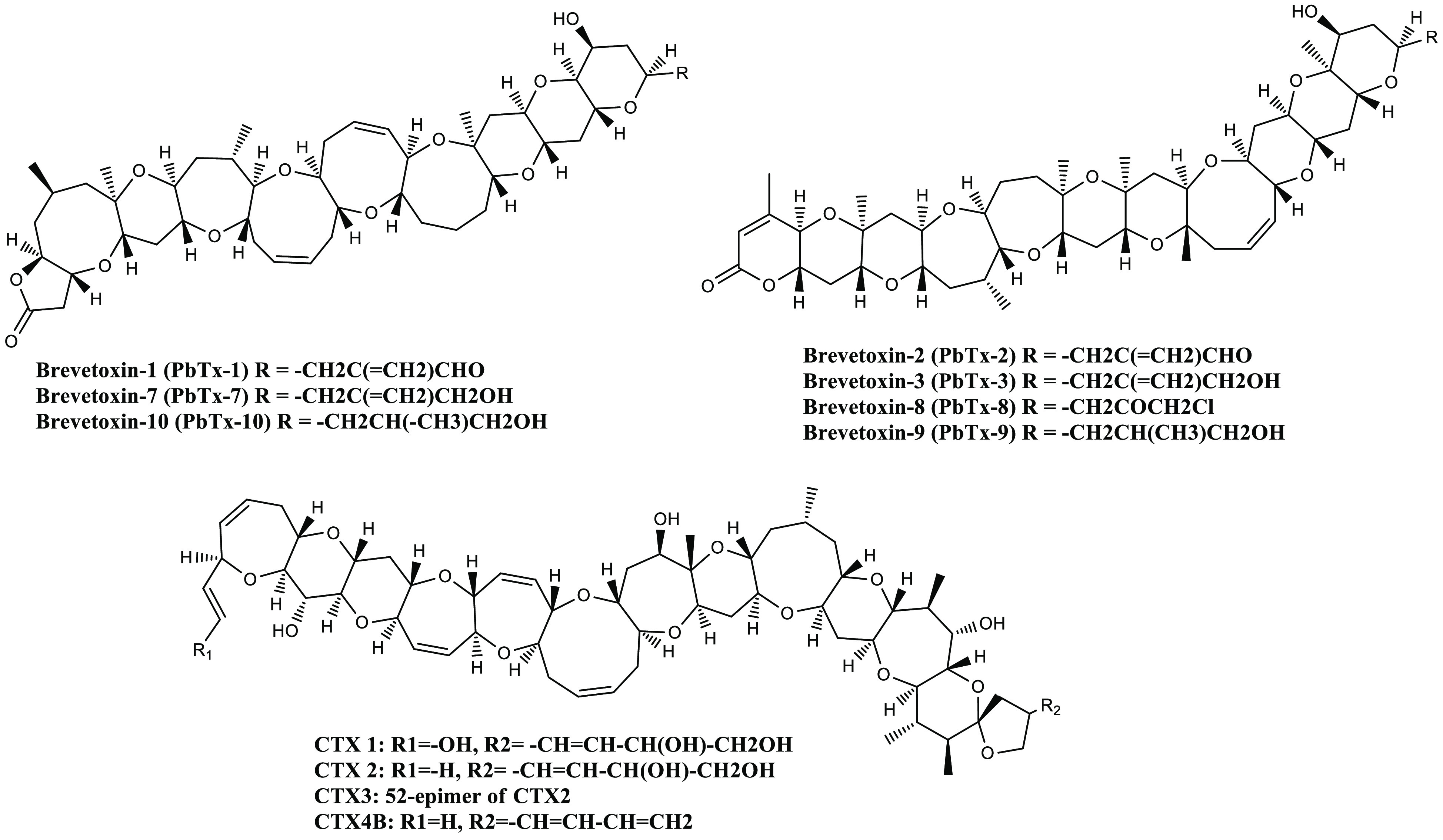
General structures of various marine biotoxin (Brevetoxin).

Neurotoxic Shellfish Poisoning (NST) is another type of algal toxin that causes neurological as well as gastrointestinal ailments. Brevetoxins are a kind of marine biotoxin produced by *Karenia brevis* (Florida red tide dinoflagellate). It is a poly(ether ladder) compound (Figure 5). It causes mortality in massive fish and marine mammals. In humans, these toxins resulted in asthma-like symptoms if inhaled.^172^ Neurological and toxicity symptoms of NST are paralysis, seizures, paresthesia, and coma, while gastrointestinal ailments are represented by nausea, diarrhea, vomiting, cramps, and bronchoconstriction, and extreme poisoning may also lead to death.^161^ Ciguatera fish poisoning (CFT) is one of the most common foodborne illnesses caused by marine biotoxin of ciguatoxin.^173,174^ Ciguatoxins are toxic and lipid-soluble compounds found in marine organisms. Gambiertoxins, the precursor toxins, are produced by benthic dinoflagellates of *Gambierdiscus* genus. These toxins are accumulated in large predatory fishes like Spanish mackerels, moray eels, barracuda, and snappers.^161^ These compounds abnormally activate sodium ion channels and disrupt the cell membrane.^175^ These compounds cause abdominal pain, nausea, diarrhea, vomiting, hypertension, and bradycardia along with neurological complications.^161^

#### 4.3.1 MS Analysis and Detection of Algal and Marine Biotoxins

Algal and marine biotoxins are another class of heterogeneous toxins produced by algae and cyanobacteria, Dinoflagellates and diatoms produced during algal blooms in rivers, freshwater lakes, and marine aquatic systems.^176^ Karunarathne et al., have found that 16,659 deaths have been reported in India between 1999 and 2018 due to poisoning.^177^ In the case of sea food such as shellfish, exposure and contamination to multiple toxins are possible, hence an efficient system is able to detect diverse classes of toxins at the same time effectively. Blay et al.,^178^ developed a method for the detection of multiple lipophilic biotoxins including azaspiracids, dinophysistoxins, and pectenotoxins as well as negative toxins via reversed-phase LC–MS within 7 min and hydrophilic toxins such as okadaic acid, dinophysistoxin-1,2, and yessotoxin from shell fish by recording scans at 2 Hz in positive and negative scans alternatively and 1 Hz in positive mode, respectively. Hydrophilic toxins including gonyautoxins domoic acid and saxitoxin were detected with mass accuracy of less than 1 ppm error and resolving power of 100,000 for the analytes (*m*/*z* 300–500). The limits of detection for lipophilic toxins were 0.041–0.10 μg/L ppm (positive ions), 1.6–5.1 μg/L (negative mode), and 3.4–14 μg/L for domoic acid and paralytic shellfish toxins.^178^ The biggest advantage of the method is that the analytes were detected with real time samples without any interference. Aquatic water bodies have a higher possibility of having aquatic biotoxins; hence, monitoring of water in water bodies is mandatory. Estevez et al. developed a method to seawater monitoring for marine biotoxins by hydrophilic interaction liquid chromatography coupled with HRMS. The main analytes considered for the detection were saxitoxin, decarbamoyl-saxitoxin, neosaxitoxin, gonaytoxin-2,3, and tetrodotoxin due to their adverse effects on gastrointestinal and central nervous systems in humans if taken up via seafood. Samples were processed via ultrasound-assisted solid–liquid extraction with methanol to extract toxins, followed by solid phase extraction using silica cartridges. The selected toxins are polar in nature; hence, the extraction stage is crucial for analysis, and the developed method has recoveries of 15–47% in filtrate and 26–71% in particulate fraction. Simultaneously, limit of detection was also affected with source as LOD was 0.5–5 μg/L for filtrate and 3.1–62 μg/L for particulate fraction.^179^

Kolrep et al.^180^ conducted comparative metabolite profiling to track the metabolism of okadaic acid in the liver and the role of CYP3A4 and CYP3A5 in its detoxification. It was found that LC–MS/MS can identify the metabolites distinctly from humans and rats based on the difference in +16 (+O) and +14 (+O/–H_2_) Da. It suggested some critical differences in the metabolism of okadaic acid in humans and rats. In continuation, it was also found that rats generated more metabolites from okadaic acid in comparison to humans in the presence of NADPH-dependent enzymes.^181^ The establishment of metabolic patterns and fragments might be crucial for the identification of fingerprints for toxin identification. Table 5 elaborates on the detection of bacterial and marine biotoxins from different samples.

**Table 5:** Detection of Bacterial and Marine Biotoxins by MS-Based Approach

Name	Sample	Method	Operating parameters	Outcome	Ref
Enterotoxin	Commercial	LC–HRMS	LC–MS Q-extractive mass spectrophotometer C18 reverse phase column; isocratic elution	93 signature peptides identified for enterotoxins	(182)
Botulinum	Commercial	Endopep MS	Triple quadrupole mass spectrometer; Turbo Ion Spray interface; C18 column; gradient elution	Toxin detection 0.1 MLD_50_ and quantification 0.62 MLD_50_	(183)
Okadaic acid	Raw and cooked food (Mussel, clam, flatfish)	Tandem mass spectrometry	C18 column with triple-quadruple mass spectrometer; ammonium format gradient elution; negative ionization mode	LOD and accuracy 0.2–5.1 μg/k	(184)
Dinophysistoxin	Raw and cooked food (Mussel, clam, flatfish)	Tandem mass spectrometry	C18 column with triple-quadruple mass spectrometer; ammonium format gradient elution; negative ionization mode	LOD and accuracy 0.2–5.1 μg/k	(184)
Anatoxins a (ATX) and Homoanatoxin-a (HAT)	Benthic-cyanobacterial-mat field samples	LC–HRMS/MS	Q Exactive HF Orbitrap MS; HESI-II electrospray ionization source; at 40 °C; resolution 60,000; collision energy 20 eV	Toxins are in conjugated form 15% ATX and 38% HAT	(185)
Cyanotoxins	Blue-green algae dietary supplement	Hydrophilic Interaction Liquid Chromatography-Tandem Mass Spectrometry	Electrospray ionization positive; source temperature 550 °C; ion spray voltage 5500 V; curtain gas 25 psi; collision gas 10 psi	Quantification limits 60–300 μg kg^–1^	(186)
Paralytic shellfish toxins	Marine shellfish	Hydrophilic interaction chromatography-tandem mass spectrometry	Separation with HILIC-Z column; acetonitrile and ammonium formate-formic acid as the mobile phase; positive electrospray ionization; samples are cleaned with by ion-pair SPE using a porous graphitic carbon cartridge	Limits of detection 1.7–13.7 μg kg^–1^; and quantitation 5.2–41.0 μg kg^–1^; recoveries 76.5–95.5%	(187)
Tetrodotoxin	Marine shellfish	Hydrophilic interaction chromatography-tandem mass spectrometry	Separation with HILIC-Z column; acetonitrile and ammonium formate-formic acid as the mobile phase; positive electrospray ionization; samples are cleaned with by ion-pair SPE using a porous graphitic carbon cartridge	Limits of detection 1.7–13.7 μg kg^–1^; and quantitation 5.2–41.0 μg kg^–1^; recoveries 76.5–95.5%	

### 4.4 Phytotoxins

**Figure 6:**
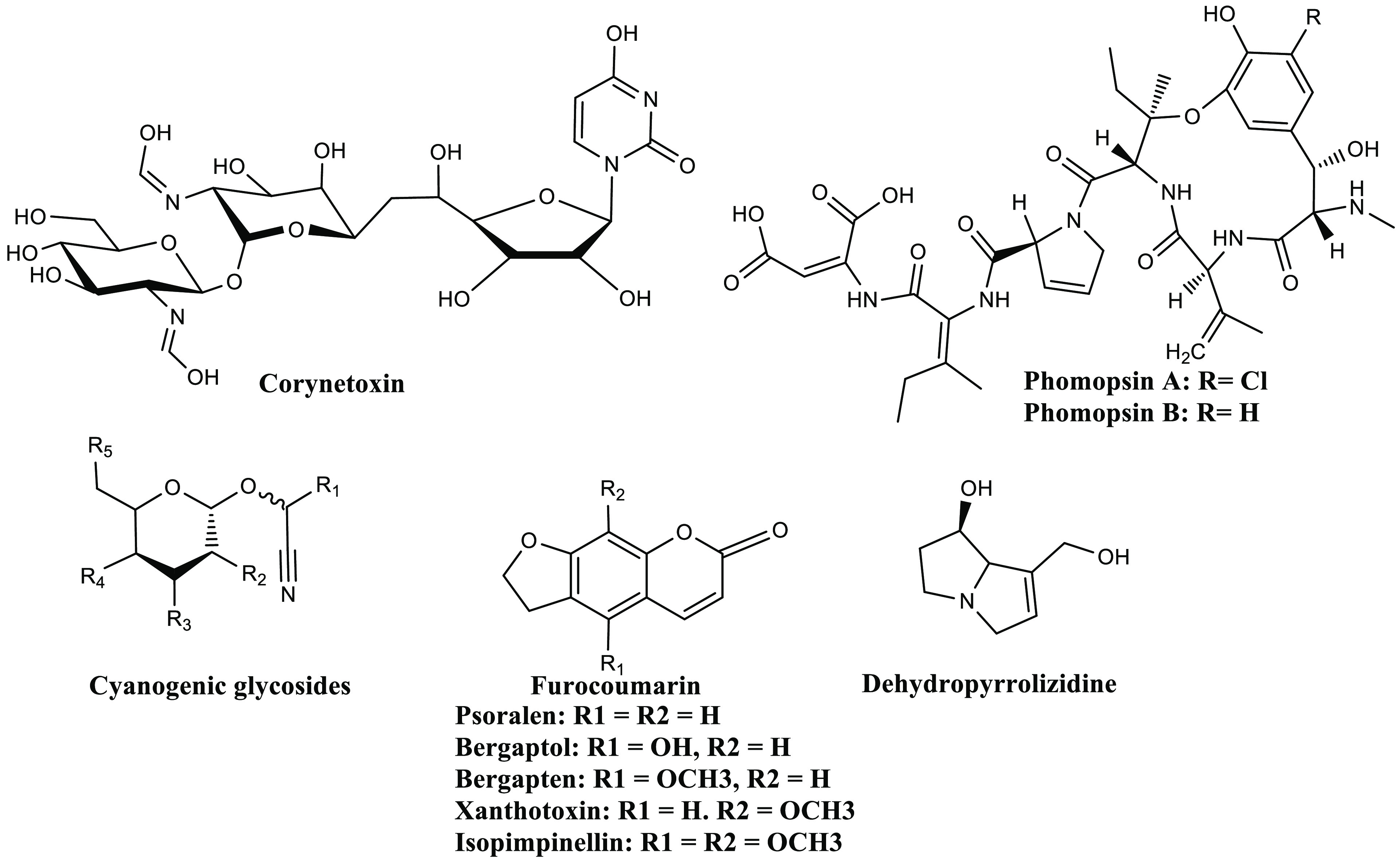
General structures of various Phytotoxins. The general scaffold for Cyanogenic glycosides, Furocoumarin and Dehydropyrrolizidine, has been depicted with providing a few examples of various compounds belonging to the class of Furocoumarin.

Phytotoxins are plant-derived compounds, including alkaloids and glycoalkaloids, that are naturally produced within plants but prove harmful if they remain in food products (Figure 6). These are secondary metabolites in plants and include cyanogenic glycosides, glucosinolates, glycoalkaloids, pyrrolizidine alkaloids, and lectins.^188^ Based on the site, these toxins can be classified into endotoxins and exotoxins. Endotoxins may be normal metabolites that are present in cells but become harmful if consumed in higher concentrations, and these compounds are also refereed as antinutritional factors, while exotoxins are toxic metabolites that are released from cells. Based on chemical nature, these are dehydropyrrolizidine, alkaloids, ptaquiloside, corynetoxins, and phomopsins.^189^ Cyanogenic glycosides (CGLs) are present in almonds, cassava, bamboo roots, sorghum, and stone fruits. These toxins are generated from proteinogenic amino acids like leucine, isoleucine, phenylalanine, tyrosine, and valine as well as nonproteinogenic amino acids like cyclopentenylglycin. CGLs are potentially toxic for humans and result in acute cyanide intoxication, high respiration rate, lower blood pressure, headache, dizziness, stomach pains, diarrhea, vomiting, and mental confusion.^188,189^ Furocoumarins are found in many plants including carrots, celery roots, citrus fruits, parsley, and citrus plants. These compounds are responsible for gastrointestinal ailments and phototoxicity, skin reactions under UV light.^34,190^ Lectins are reported from beans like kidney beans and can result in stomachache, diarrhea, and vomiting.^189^

Phytotoxins are sometimes a part of the natural defense of plants, like Pyrrolizidine alkaloids (PAls) that are produced in *Asteraceae*, *Boraginaceae*, and *Fabaceae* families to defend plants against herbivores as well as insects. These toxins tend to have a common 1-hydroxymethyl pyrrolizidine core that is esterified with aliphatic acids. Besides edibles from these plants, honey is one of the common products contaminated with PAls, and hence, the extract or infusion can be used as an analyte for the detection of PAls. Ten plant samples, *Anchusa officinalis*, *Borago officinalis*, *Echium italicum*, *Eupatorium cannabinum*, *Heliotropiumeuropaeum*, *Lithospermum officinale*, *Petasites hybridus*, *Senecio vulgaris*, Symphytum officinale, and *Tussilago farfara* from Orto botanicodellaScuola Medica Salernitana, Salerno, Italy, were collected, and aqueous extract was prepared with salting-out assisted liquid–liquid extraction. The aqueous extracts were analyzed with UPLC–MS/MS. The analysis was able to identify 88 PAs from 282 samples with an identification limit of 0.6–30 μg kg^–1^ and a false negative rate <1.3% (at the concentration range of 4 μg L^–1^).^191^

For simultaneous detection of phytotoxins and microbial toxins, HRMS was employed, which applied to over 156 compounds inclusive of about 90 plant toxins (e.g., various alkaloids and aristolochic acids), about fifty-four mycotoxins, and 12 phytoestrogens (e.g., lignan, isoflavones, coumestans, etc.) in plant-protein samples, like cereals. MS library, created on fragmentation pattern obtained with both negative and positive ionization modes for each toxin, using ten different collision energies was used for analysis. A typical workflow was followed with generic QuEChERS-like sample preparation, followed by UPLC using suitable mobile phases that allowed the resolution of over 50 toxic alkaloids. The method performance was evaluated for its sensitivity at levels ranging from 1–100 μg/kg, and reproducibility. The quantitation obtained against the standard addition approach could meet SANTE/12682/2019 criteria for 132 toxins out of the tested 156 toxin samples.^192^ The plant toxins ricin and RCA120 were detected, differentiated, and quantified by Kalb et al.,^193^ via MS-based methods for the EQuATox proficiency test, in ∼9 samples. They successfully identified the samples spiked with ricin or RCA120; samples spiked with a 0.414 ng/mL concentration could not be detected. Liang et al.^194^ employed reversed phase LC–HRMS to detect five major phytotoxin groups including alkaloids, aromatic polyketides, flavonoids and steroids, and terpenoids at alkaline pH (>9). The developed method not only allowed the detection of 30 phytotoxins but also had forty-times higher detection sensitivity in comparison to older methods. Table 6 discusses some of the examples for phytotoxins detection using the MS-based approach.

**Table 6:** Phytotoxins Detection from Food Samples via MS-Based Approach

Name	Sample	Method	Operating parameters	Outcome	Ref
Toxoflavin and Fervenulin	Food samples and *Tremella fuciformis* Berk	UPLC–MS/MS	Separation column: ZORBAX SB-C18 column; oven temp: 35 °C; mobile phase: 0.1% formic acid+ methanol; flow rate 0.4 mL min^–1^	Detection limits (μg/kg) Toxoflavin 12	(152)
				Fervenulin 24 Toxin level (mg/kg) Toxoflavin: 7.5; fervenulin: 3.2	
Ricin	Soft drinks and serum	Surface-assisted laser desorption/ionization mass spectrometry	Pulsed Smartbeam II 2 kHz laser; wavelength 355 nm (∼3.49 eV); frequency 1000 Hz; delayed extraction time 350 ns for proteins	Limit of detection 0.5 pmol/μL	(195)
Pyrrolizidine Alkaloids	LC–MS/MS	Tea	UPHPLC with Quadrupole mass spectrometer; C18 Hypersil Gold column fitted; gradient elution	Total PA levels 13.4 to 286,682.2 μg/kg d.m	(196)
Ptaquiloside	LC–MS/MS	Bracken fern	LC–MS/MS; C18 column; gradient elution; column temperature 35 °C; electrospray positive ionization	Limits of detection 0.03 and quantification 0.09 μg/kg	(197)
Furanocoumarin	UPLC–MS/MS	*Citrus* sp.	Nexcol C18 column; column temp 40 °C; gradient elution; positive electrospray ionization	Compound recovery 94.07–114.53%	(198)
Amygdalin	LC–MS/MS	Kernels and Almonds	LC–MS/MS equipped with 6500 quadruple linear ion trap (QTRAP) mass spectrometer and electrospray ionization	Recovery 90–107%; limit of detection 0. Ng/g and limit of quantitation 8 and 2.5 ng/g	(199)
Pyrrolizidine alkaloids	quadrupole orbitrap MS	Honey	Polar C18 column; temp 40 °C; mobile phase flow rate of 400 μL min^–1^	Limit of quantification 0.1–0.3 μg kg^–1^	(200)
			MS detection with positive ionization mode; scan range 250–500 *m*/*z* and 70 k (fwhm)		
Pyrrolizidine alkaloids	quadrupole orbitrap MS	Black and green tea	Polar C18 column; temp 40 °C; mobile phase flow rate of 400 μL min^–1^	Limit of quantification 1–11.7 μg kg^–1^	(200)
			MS detection with positive ionization mode; scan range 250–500 *m*/*z* and 70 k (fwhm)		
Pyrrolizidine alkaloids	quadrupole orbitrap MS	Herbal infusion	Polar C18 column; temp 40 °C; mobile phase flow rate of 400 μL min^–1^	Limit of quantification 0.9–2.1 μg kg^–1^	(200)
			MS detection with positive ionization mode; scan range 250–500 *m*/*z* and 70 k (fwhm)		

### 4.5 Emerging Toxins

In addition to conventional toxins already known and summarized above, there is a group of toxic chemicals that are continuously evolving, mainly due to rising pollution. In the last eight years, the list of emerging chemicals has increased day by day. Synthetic chemical toxins include microplastics, organophosphorus and polybrominated flame retardants, perfluoroalkyl compounds, food process and packaging, waste substances, and nanomaterials.^15^ Besides, heavy metals, antibiotics and drug traces and metabolic intermediates, and agricultural chemicals^201^ have shown bioaccumulation and have serious toxic effects if consumed, even in low concentrations. Health ailments include endocrine disruption, suppression and overexpression of the immune system, inflammation, abnormal metabolic changes, skin diseases, carcinogenesis, etc., and the toxicity relies on interaction with the cellular system and receptors.^15,201,202^

With the increase in pollution and intrusion of pollutants in the food web, the toxic chemicals traced in food and edibles are increasing. Some of those chemicals exhibited bioaccumulation and become silent killers, but some are potentially lethal. These emerging pollutants include pesticides, herbicides, healthcare, cosmetic chemicals, etc. Fipronil is a wide-spectrum phenylpyrazole insecticide used to control beetles, ants, cockroaches, etc. but its entry into the food chain is alarming due to its carcinogenic nature, essentiating its prohibition by the US Environmental Protection Agency (EPA). Suitable detection methods are thereby essential to identify and quantify these contaminants before they enter the food chain. The most reliable detection method includes LC–MS/MS and GC–MS, having specific sample preparation before the analyses (Table 7).^203^ One such preparatory method involved a modified QuEChERS sample preparation before using a triple quadrupole MS instrument coupled to ESI for detecting fipronil and its major metabolite fipronil sulfone, at concentrations of 5 μg/kg. The use of nontargeted approaches, such as SWATH-MS (sequential window acquisition of all theoretical mass spectra), enables the sequential analysis of fipronil and other such contaminants, e.g., pesticides and polyaromatic hydrocarbons. Glyphosate (insecticide) was detected in an underivatized form by innovating new extraction methods coupled with instrumentation. The QuPPe (Quick Pesticide Preparation) method was used for sample preparation^204^ followed by detection via sensitive MS instruments to achieve accurate quantitative results. LC–MS/MS was used in combination with the DMS (differential mobility separation) technique to terminate analytical interferences leading to improved signal by decreasing noise and, consequently, increasing accuracy and confidence in data. Using this method, LC–DMS–MS/MS was used for identification and quantification of pesticide contaminants in food samples. Triclosan is a well-known and common biocide agent against bacteria as well as fungi,^205^ while bisphenol analogues are used in packaging and lining.^206^ Morgan et al.^206^ employed GC–MS to monitor the levels of triclosan and five bisphenol analogues (B, F, P, S, and Z) in 776 adult solid food samples. More than 80% of the samples were contaminated with at least one target phenol. Based on the frequencies, 59% of samples were contaminated with triclosan followed by 32% bisphenol S, and 28% bisphenol Z. The maximum concentration for triclosan was 394 ng/g.

**Table 7:** Detection of Emerging Toxins from Food and Water Samples by MS

Name	Sample	Method	Operating parameters	Outcome	Ref
Cypermethrin	Baby food	liquid chromatography coupled to quadrupole Orbitrap mass spectrometry	Gradient elution; negative ionization mode; capillary temperature 300 °C and heater 305 °C	Detection concentration in baby food 10.3 μg kg^–1^	(208)
Parabens	Surface water	UHPLC–MS/MS	LC-18 column; column temp 40 °C; gradient elution; capillary voltage −3.0 kV	Limit of detection 0.04 ng L^–1^ and Limit of quantification 0.82 ng L^–1^	(209)
Bisphenol	Water	GS–MS/MS	Temperature transfer line 250 °C; ion source 230 °C and quadrupole 150 °C. solvent delay 4.5 min; electron ionization (EI) mode (70 eV)	Recovery 81.8% −96.1%; limit of detection was 0.2 ng L^–1^	(210)
Parabens	water	GS–MS/MS	Temperature transfer line 250 °C; ion source 230 °C and quadrupole 150 °C. solvent delay 4.5 min; electron ionization (EI) mode (70 eV)	Recovery 81.8% −96.1%; limit of detection was 0.2 ng L^–1^	(210)
Triclosan	water	GS–MS/MS	Temperature transfer line 250 °C; ion source 230 °C and quadrupole 150 °C. solvent delay 4.5 min; electron ionization (EI) mode (70 eV)	Recovery 81.8% −96.1%; limit of detection was 0.2 ng L^–1^	(210)
Neonicotinoids	vegetables	QuEChERS-Portable MS	PDESI as ion source; ultrapure helium (≥99.999%) as carrier gas; inlet temperature 200 °C; molecular pump speed was 1375 Hz	Limit of detection 2.0 ng g^–1^ recovery 82.2% −109.7%	(211)
Carbamates	Vegetables	QuEChERS-Portable MS	PDESI as ion source; ultrapure helium (≥99.999%) as carrier gas; inlet temperature 200 °C; molecular pump speed was 1375 Hz	Limit of detection 2.0 ng g^–1^ recovery 82.2% −109.7%	(211)
Phenyl Pyrazole	Vegetables	QuEChERS-Portable MS	PDESI as ion source; ultrapure helium (≥99.999%) as carrier gas; inlet temperature 200 °C; molecular pump speed was 1375 Hz	Limit of detection 2.0 ng g^–1^ recovery 82.2% −109.7%	(211)
Pesticides	Fruits and vegetables	QuEChERS-LC–MS	Sciex QTRAP 5500 triple quadrupole MS; positive electrospray ionization; ion source temperature 550 °C	24 pesticides detected distinctly	(212)
Pesticides	Milk	LC-LTQ/Orbitrap Mass Spectrometry	Separation with reverse phase C18 column; positive ionization mode; spray voltage 4 kV; auxiliary gas flow rate 10 arbitrary units; tube lens 90 V, capillary temperature 320 °C	Limit of detection 0.2–8.1 μg kg^–1^ and quantification 0.61–24.8 μg kg^–1^	(213)

Not only emerging toxins but also the availability of efficient and portable systems have become necessary prerequisites. Some of the recent advancements have shown the availability of portable MS systems for detection and monitoring. Maragos^131^ has evaluated the potential of portable MS (APCI-MS) for the detection of T-2 toxin mycotoxin in contaminated cereal grains, wheat, and maize by APCI-MS. The sample was extracted with acetonitrile+water (84:16, v/v) followed by drying and reconstitution in ammonium formate. The MS system contains a linear ion trap mass analyzer to avoid the need of an external supply of gas or air. The device and developed method were able to detect T-2 toxin above 0.2 mg/kg from soft white and hard red wheat, and yellow dent maize. The method was more efficient and hence able to lower-down the detection limit from >0.9 mg/kg. In a similar line, FB and its isoforms were detected in maize with a portable mass spectrometer. For the detection, samples were extracted with aqueous methanol followed by cleaning up in the immunoaffinity column. Ultimately, cleaned samples were successfully analyzed with the portable MS with detection limits of 0.15 (B1), 0.19 (B2/B3), and 0.28 (total FB) mg/kg maize. The method has quantification limits of 0.33, 0.59, and 0.74 mg/kg and recoveries of 93.6% to 108.6%. Wichert et al.^207^ have also reported such kind of advancements to detect proteinaceous toxins (912.5–66.5 kDa) from plants as well as microorganisms origin using paper spray-MS (PS-MS) with wipe samples of bench, glass, leaves, flooring, etc., and validated with biological toxin simulant for *Staphylococcal* enterotoxin B. Carbon sputtered porous polyethylene dominated conventional chromatography paper, carbon nanotube-coated paper, and polyethylene for paper spray. The method was able to distinguish the protein toxin simulant efficiently with a good signal-to-noise ratio.

## 5 Detection of Food Fraud and Food Adulteration

Food adulteration and fraud have become a common practice nowadays to gain more profit. To avoid detection by current available analytical methods, new adulteration and fraud practices are becoming advanced and sophisticated making detection one of the biggest challenges for society.^214^ The tracing of fraud in food products via chemical analysis has become more complex especially due to the emergence of new and unknown adulterants.^74^ MS has become an indistinguishable part of testing and food authentication analysis due to its potential to trace chemical compounds based on their chemical fingerprints or chemical profiles.^215^ Adulterants are used as ingredients and additives in food products for economic gain for the seller at the cost of the health of consumers. The use of bulking agents such as sulfated polysaccharides is very common in minced meat and is a fraudulent practice that is very difficult to detect. Kosek et al.^216^ have evaluated rapid evaporative MS (REIMS) to detect adulterants in sausages and burgers prepared from chopped pork and chicken meat. The technique was able to detect the adulterants efficiently with 2.5% as the threshold concentration for protein additives (carrageenan) can be detected at 1% concentration. The major advantage of the system is the quick detection of adulterants in samples, which aids in preventing any major health issue from the consumption of adulterated food products. The problem becomes more serious when consumers are infants or minors. Milk is one of the common products that is supposed to provide optimum nutrients including lipids and proteins. Piras et al.^217^ employed atmospheric pressure matrix-assisted laser desorption/ionization with a Q-TOF mass analyzer, which generated charged proteinaceous as well as lipids/metabolites ions to find the possible fraud with milk. The common adulteration in milk is the intermixing of milk from different sources, and the current methods were able to identify milk from camel, cow, goat, and sheep with 100% accuracy. The goat milk was analyzed for the presence of cow milk as an adulterant, and it was detected even at a lower concentration of 5% with sensitivity and specificity of 92.5% and 94.5%, respectively. The major outcome of the work is its time consumption for sample analysis, i.e., 10 s per unadulterated sample to prepare profile. The method creates differences between protein and lipid molecules in terms of the number of charged moieties as lipids are single charged, while protein moieties have multiple charges.

Integration of artificial intelligence and neural network models has further improved the demand for efficiency of MS systems. Nichani et al.^218^ have developed a method for the differential identification of spelt and wheat. Nontargeted LC–MS/MS along with convolutional neural network (CNN) models was developed. The employed neural network was able to learn patterns by itself and discriminated between spelt and wheat efficiently. For external validation of the model, artificial mixed spectra of spelt bread and flour, 11 untypical spelt, and six old wheat cultivars (which were not part of model training) were analyzed. The model was able to identify the nonconventional cultivars of wheat and spelt with a *D* value of 0.57.^218^

Not only food products but also active pharma formulations and ingredients are under the threat of adulteration and fraud. One such example is where MS is used to develop a model to differentiate between wild and cultivated *Cordyceps sinensis* harvests. Elemental analysis coupled with stable isotope ratio MS (EA-IRMS) and GasBench II coupled to isotope ratio MS (GB-IRMS) was used with orthogonal partial least-squares discriminant analysis (OPLS-DA) to identify the significant and unique markers of stable isotope ratios in both samples. Analysis identified three stable isotope markers, i.e., δ2H, δ18O, and δ15N, and their concentrations can be used to identify fresh *C. sinensis* samples as well as differentiating between wild and cultivated *C. sinensis*. δ2H values reduced sequentially in fresh samples based on respective origins. δ18O and δ15N have shown differential patterns as lower δ18O and higher δ15N represented cultivated samples, while higher δ18O and lower δ15N represented wild samples. The analyzed cultivated and wild samples have δ18O and δ15N of 18.99%, 4.80%, and 25.72%, 2.29%, respectively.^219^

## 6 Current Challenges and Future Prospects

The methods currently in practice for toxin detection are quite advanced, comprising a variety of direct and indirect analytical techniques.^220^ However, there are still a few challenges that make laboratory detection difficult, and toxins may sometimes go undetected.^221^ The following are the significant challenges for toxin analysis that are commonly encountered.

### 6.1 Representative Sample

Microbial toxins are rarely evenly distributed in foodstuff and edibles.^222^ As in the case of mycotoxins, in some regions of the victuals, called “mycotoxin pockets”, concentrations may be extremely high, whereas the rest of the material may be free from contamination. The materials are distributed more heterogeneously for products with larger particle sizes, such as nuts and figs. This necessitates representative sampling that accounts for the random distribution of the “hotspots” to provide an accurate view of the degree of contamination in the specimen. This can be performed by taking a large number of small, incremental samples from various locations distributed throughout the lot.^223^ The selection of incremental samples from the bulk is crucial to give all morsel particles a chance of being selected, thus reducing the statistical bias. Besides the number of samples, sample type and its processing are equally important as in some cases surface swab is sufficient, while others need extraction followed by processing like digestion of target sample as reported in the case of protein-based toxins. Kalb et al. employed endopep-MS to identify botulinum A, B, E, and F from food samples by optimizing the fingerprint peptides generated.^149^ Hence, detection technology must be optimized to compete with emerging challenges.

### 6.2 Sample Preparation

Sample preparation is a highly complex process, with various pitfalls. Every step encountered introduces a level of variability that aggregates and contributes to total variability within single analytical data. It has been generally observed that nearly 1/3 of the variability is attributed to sample preparation. On the other hand, a much smaller amount of variation is contributed by the analytical method being employed. Even with the best analytical equipment, sample preparation is critical.^224,225^ By adhering to the key factors of sample preparation (size reduction, sampling size, and uniformity), the root–mean–square value can be kept significantly within 5–10%, increasing the prediction result’s accuracy.

### 6.3 Low Concentration

Even at low concentration levels, toxins can be highly toxic.^226^ Different classes of toxins have different levels of toxic effects on the target. Glycoalkaloids (potato) and isoflavones (clover) have shown low toxicity, linamarin (cassava) and coniin (hemlock) are somewhat toxic, while ricin (castor beans) and cyanotoxin and saxitoxin (blue–green algae) are extremely toxic.^227^ Hence, the tolerable concentration range is also varied in the same proportion. The Food Safety and Standards Authority of India (FSSAI) has set limit values of 15 μg/kg in cereal products, pulses, and nuts, and 30 μg/kg in spices, whereas, for milk, the allowable range is considerably low (0.5 μg/kg).^228^ The values are more stringent for the US FDA and European Union, nearly 1/3 of the FSSAI-approved figures. The analysis method needs to be extremely precise and sensitive to detect such low concentrations. The low levels of toxin concentration, often not detected, are hugely responsible for reduced production efficiency and increased susceptibility to various diseases.^229^

### 6.4 Complex Matrix

Generally, the toxin matrices found in food are fairly complex and pose a major challenge for laboratories. For example, heterogeneous matrices such as spices contain numerous interfering substances, which makes it extremely difficult to detect the toxins precisely. Also, food may not be necessarily safe, even if well-known toxins are not detected during analysis. These compounds may still be present in conjugated form in masked or bound form.^230^ These modified toxins are derived from plants by conjugation and have their chemical structures altered, making the analysis more complex. Also, even though a large number of different toxins including mycotoxins, bacterial toxins, phytotoxins, etc. exist, only a few are characterized and regulated by law.^2^ In reality, several toxins may be present simultaneously, and it may be challenging to find/pinpoint specific analytes in the food particles.

### 6.5 Portability of Instrument

The normal trend in sample analysis is like the collection of samples from the site and transfer to the lab for further analysis. However, such a protocol seems obsolete when we need to characterize the sample rapidly. In such cases, a portable and handy instrument with easily transferable and ready-to-use techniques is required. Some of the recent research has shown the pay for portable MS-based detection systems for the food analysis and characterization of toxins like PS-MS.^207^ However, more advancement needed with the miniaturization of instruments with rapid detection and high accuracy is required.

### 6.6 Cost

A variety of factors contribute to the cost of toxin analysis. They include facilities, sophisticated analytical instruments, reagents, and logistics. Additionally, the number of tests needed for representative sampling to negate the misreporting of toxins in bulk material also contributes to the testing cost. Although effective sampling is one of the most critical factors in mycotoxin analysis, it is the costliest, and surprisingly, innovations in this area have not taken place rapidly.^231^ Reducing the time and cost requirements through representative sampling while increasing accuracy is the need of the hour. A balance needs to be achieved between the cost per sample and the number of runs essential to generate the most confident results that will ensure lower downtime with precise results.^232^

### 6.7 Multiple Toxins Contamination

Naturally, all the food materials are prone to be contaminated with multiple toxins due to the presence of diverse microbial contaminants simultaneously. The method should be effective and efficient to detect all of the toxins together even in minute quantity. A MS-based approach made it possible to allow the detection of diverse range of toxins. Lattanzio et al.^233^ have reported the detection of multiple aflatoxins including B1, B2, G1, G2, ochratoxin A, fumonisins B1 and B2, deoxynivalenol, zearalenone, T-2, and HT-2 toxins simultaneously from maize via LC–MS. In a similar line, Cheng et al.^234^ have also reported the detection of 15 toxic alkaloids from vegetables and meat samples using double layer pipet tip magnetic dispersive solid phase extraction method that used polyamidoamine-functionalized magnetic carbon nanotubes. Extracted samples were characterized with ultrafast liquid chromatography-tandem quadrupole mass spectrometry (UFLC–MS/MS) coupled with DPT-MSPE method. The system has shown high recovery efficiency with toxin recovery of up to 125% for meat as well as vegetable dishes. In food samples, fungal contamination is a common phenomenon; hence, it is the prime target for most of the work. Wang et al.^235^ and Nualkaw et al.^236^ have employed UPLC–MS for the detection of mycotoxins from animal feed like dairy product, poultry, and animal feed. Lee et al.^184^ reported the presence of okadaic acid, dinophysistoxin-1, dinophysistoxin-2, and dinophysistoxin-3 in raw as well as cooked mussels using LC–tandem mass spectrometry. The method has shown detection and quantification limit of 0.2–5.1 μg/kg with accuracy and precision of 80.5–109.8% and 0.9–20.1%, respectively. Albero et al.^237^ have also identified mycotoxins in aquaculture feed by LC–MS/MS. The method employed ultrasound-assisted extraction followed by LC–MS/MS, which identified 15 mycotoxins together that also included enniatins (EENB and ENNB1), beauvericin, and fumonisin B2.

In some cases, the toxins are present in modified or bounded form (masked form), which hinder its detection by conventional methods. As mentioned by Berthiller et al.,^238^ mycotoxins have high probability of masking due to food processing which changes its structural as well as behavior and obstruct the appearance of common characteristics of respective toxins. Masked mycotoxins have been detected in extractable conjugated and nonextractable varieties (usually to unavailability of toxins in extracted samples, bound mycotoxins are not accessible for analysis and need chemical or enzymatic treatment prior detection).^238^

Fiby et al.^239^ reported the presence of *Fusarium* mycotoxin like deoxynivalenol in native as well as masked form (DON-3-glucoside “D3*G*”, 3-acetyl-DON “3ADON”, or 15-acetyl-DON “15ADON”) in cereals. The presence was detected with a stable-isotope dilution liquid chromatography–tandem mass spectrometry-based approach that relies on labeling of toxins enzymatic byproducts with 13C. The method efficiently detected D3G (76–98%), DON (86–103%), 15ADON (68–100%), and 3ADON (63–96%).^239^ Zhang et al.^240^ employed ultrahigh-performance liquid chromatography–HRMS to detect 82 mycotoxins and categorize into 8 classes by Python program developed with “Fragmentation pattern screener (FPScreener)” and nontarget screening rules. Pascari et al.^241^ combined QuEChERS with liquid chromatography–triple quadrupole mass spectrometry for the detection of ZEN from oat flour. For the identification, oat and wheat flours were treated with amylolytic enzymes (α-amylase and amyloglucosidase), similar to the one used in the cereal-based baby food production process that reduced the β-zearalenol (β-ZEL) and β-ZEL-14-sulfate by 40% within 90 min and allowed the specific detection of ZEN-sulfate derivates from cereals.

## 7 Future Perspective

The past few decades have witnessed significant advancements in analytical techniques and technologies to detect and manage the problem of mycotoxin analysis. The global mycotoxin testing market is estimated to grow at a cumulative annual growth rate (CAGR) of 7.8%, which corresponds to a market of $1.4 billion by 2026.^242^ As the number indicates, the accelerated need for food safety has significantly accelerated the mycotoxin testing market. Needless to say, effective sampling, method performance, cost, and rapid detection are of paramount importance for successful mycotoxin testing. However, inexpensive and fast tests with low precision would lead to misclassification through inaccurate results and impact the business decisions of the producers. In light of this, the existing techniques must be refined/tuned for higher precision while active R&D is pursued to develop new methods that can detect multiple toxins simultaneously with high sensitivity (at regulatory levels) with minimal cost and runtime. One option could be developing miniaturized MS, which could be used in a fashion similar to the screening devices used for COVID detection at airports and train stations. The spectrometers are generally efficient and reliable and allow for rapid detection and solid sample detection through thermal absorption. One such example is mycotoxin detection in milled wheat by an MS developed by M/s BaySpec Inc.^243^ The portable device could detect even ppm (1.4) levels of the contaminant within less than a minute’s time.

Recent studies have highlighted that mass-sensitive microarray (MSMA)-based biosensors could be a promising tool for rapidly detecting mycotoxin material.^244^ The device consists of a mass-sensitive transducer based on solidly mounted resonator (SMR) technology with a specific integrated circuit. This technology allows small molecular weight toxins (up to 3 toxins simultaneously) to be rapidly detected with high sensitivity (for a single sample) within less than 10 min using mycotoxin analysis as a model example. The authors argued that with further upgrade, many more analytes (∼32) could be detected on the devices from a single sample, and with automation, the analysis was performed and printed without the requirement of any operator. The device could be an ideal system for multiplex analysis of mycotoxins; however, significant improvements must be made before it can be considered for field deployment in the analysis of real-time samples.
